# Time-Gated Luminescent In Situ Hybridization (LISH): Highly Sensitive Detection of Pathogenic *Staphylococcus aureus*

**DOI:** 10.3390/molecules24112083

**Published:** 2019-05-31

**Authors:** Nima Sayyadi, Russell E. Connally, Thomas S. Lawson, Jingli Yuan, Nicolle H. Packer, James A. Piper

**Affiliations:** 1 Department of Molecular Sciences, Macquarie University, Sydney, NSW 2109, Australia; 2 ARC Centre of Excellence for Nanoscale Biophotonics (CNBP), Macquarie University, Sydney, NSW 2109, Australia; thomas.lawson@mq.edu.au (T.S.L.); jim.piper@mq.edu.au (J.A.P.); 3 Department of Physics and Astronomy, Macquarie University, Sydney, NSW 2109, Australia; russell.connally@mq.edu.au; 4 State Key Laboratory of Fine Chemicals, School of Chemistry, Dalian University of Technology, Dalian 116023, China; jlyuan@dlut.edu.cn

**Keywords:** homogeneous luminescent in situ hybridisation, TEGylated Europium chelate, time-gated luminescent microscopy, *Staphylococcus aureus* (*S. aureus*) detection

## Abstract

We describe simple direct conjugation of a single TEGylated Europium chelate to DNA that binds to intracellular rRNA and is then detected using a homogeneous luminescent in situ hybridisation (LISH) technique. As a proof-of-principle, *Staphylococcus aureus (S. aureus)* was selected as a model for our study to show the ability of this probe to bind to intracellular 16S ribosomal rRNA. A highly purified Europium chelate conjugated oligonucleotide probe complementary to an rRNA sequence-specific *S. aureus* was prepared and found to be soluble and stable in aqueous solution. The probe was able to bind specifically to *S. aureus* via in situ hybridisation to differentiate *S. aureus* from a closely related but less pathogenic *Staphylococcus* species (*S. epidermidis*). A time-gated luminescent (TGL) microscope system was used to generate the high signal-to-noise ratio (SNR) images of the *S. aureus*. After excitation (365 nm, Chelate λ_max_ = 335 nm), the long-lived (Eu^3+^) luminescent emission from the probe was detected without interference from natural background autofluorescence typically seen in biological samples. The luminescent images were found to have 6 times higher SNR or sensitivity compared to the fluorescent images using conventional fluorophore Alexa Fluor 488. The TEGylated Europium chelate -oligo probe stained *S. aureus* with mean signal intensity 3.5 times higher than the threshold level of signal from *S. epidermidis* (with SNR 8 times higher). A positive control probe (EUB338–BHHTEGST–Eu^3+^) has mean signal intensity for *S. aureus* and *S. epidermidis* equally 3.2 times higher than the threshold of signal for a negative NON-EUB338 control probe. The direct conjugation of a single Europium chelate to DNA provides simplicity and improvement over existing bovine serum albumin (BSA)/streptavidin/biotinylated DNA platforms for multi-attachment of Europium chelate per DNA and more importantly makes it feasible for hybridisation to intracellular RNA targets. This probe has great potential for highly sensitive homogeneous in situ hybridisation detection of the vast range of intracellular DNA targets.

## 1. Introduction

Staphylococci include *Staphylococcus aureus* (*S. aureus*) and coagulase-negative *Staphylococcus* species (CoNS). *S. aureus* is more virulent and usually causes more serious infections than CoNS [1,2,3]. The ability to rapidly and accurately distinguish between *S. aureus* and non*-S. aureus* bacteria is essential for the appropriate therapeutic use of antibiotics and timely intervention for infection control in patients. Unfortunately, after Gram staining, the definitive identification of *S. aureus* involves a number of time-consuming procedures. Traditional methods require subculturing (for 12 to 24 h) followed by biochemical analysis. Rapid identification of *S. aureus* would thus be beneficial. 

Several molecular methods such as PCR [4,5,6], nanoparticle DNA-based biosensor [7,8,9,10] and the DNA microarray approach [11,12,13,14,15] have been reported for the rapid identification of pathogenic bacteria such as *S. aureus*. However, homogeneous fluorescence in situ hybridisation (FISH) provides inexpensive and rapid identification of *S. aureus* in blood culture samples [16,17,18,19,20]. We have previously reported an improvement to the specificity of fluorescence in situ hybridisation (FISH) detection of *S. aureus*. This was achieved using a simple and effective permeabilisation technique in conjunction with a conventional fluorescent organic dye, such as Alexa Fluor 488 [21,22,23]. However, imaging the FISH labelled *S. aureus* under fluorescent microscopy has two major limitations. Firstly, the fluorescent signal from the organic dye used is rapidly quenched and loses its brightness during its excitation and visualisation. Secondly, the signal from the targeted *S. aureus* cells overlaps with the naturally high autofluorescence background found in most biological matrixes. As a result, the intensity of the FISH labelled *S. aureus* (or signal intensity) to background autofluorescence intensity ratio, that is, the signal-to-noise ratio (SNR), is dramatically reduced and consequently decreases the sensitivity of detection.

In comparison to conventional fluorescent organic dyes, lanthanide chelates have unique emission characteristics, including long excited-state lifetime, sharply spiked emission spectra and large Stokes shift. These characteristics, in combination with time-gated luminescence (TGL) microscopy, are advantageous in discriminating target signal against short lived autofluorescence in biological matrixes [24]. The high SNRs which can be achieved with lanthanide-based luminescent probes translate directly to high detection sensitivities, substantially exceeding those achievable with conventional fluorophores and making these luminescent probes an attractive alternative to radio isotope-based probes [25].

BHHCT–Eu^3+^ is a tetra-dentate *β*-diketone Europium chelate that was synthesised in our research group [26]. It has a strong luminescent emission, a high quantum yield and a long lifetime. Although BHHCT–Eu^3+^ has been used for the TGL detection of a wide range of biotargets, such as Giardia and Cryptosporidium [27], its highly reactive sulfonyl chloride group at the attachment point to biotargets gave it a rather low bioconjugate specificity. It is also highly hydrophobic and prone to precipitation when multiconjugation of chelate per immune-protein is used for bioconjugation. These limitations were addressed by the introduction of a propanyl linker that used a *N*-hydroxysuccinimide ester as the activated attachment point (BHHST–Eu^3+^)[28]. More recently, it was further modified by the replacement of the propanyl linker of BHHST with a tetra-ethylene glycol (TEG) linker that gives the resultant immune-conjugates (e.g., conjugated antibody) luminescent probe (BHHTEGST–Eu^3+^) a higher aqueous solubility and stability [29,30,31].

We have previously shown the successful use of BHHCT chelates in combination with bovine serum albumin (BSA)/streptavidin/biotinylated DNA platforms for the identification of a specific DNA sequence via luminescence resonance energy transfer (LRET) technique in luminescence in situ hybridisation fashion. Intermediate proteins, such as biotin/streptavidin pairs and BSA, were used to provide enhanced labelling of Europium chelate per oligonucleotide probe so as to increase the luminescence emission per DNA and increase detection sensitivity [32,33]. BSA was also added as an intermediate protein and increased the solubility of the BHHCT conjugate, as this chelate is inherently very hydrophobic. However, using the biotin/streptavidin platform and BSA required extra steps in its preparation, conjugation and tuning of the ratio of chelate to protein. For the same reasons, the use of a biotinylated DNA was also necessary. Even so, conjugation of multiple chelates to the intermediate reagent remained susceptible to precipitation and aggregation of the bioconjugate. More importantly, use of streptavidin/biotinylated DNA platforms for conjugation limits the application of luminescence in situ hybridisation, particularly for intracellular targets such as rRNA found in bacteria. This is because the large size of the BSA/streptavidin/biotinylated-DNA probe inhibits movement through the permeabilised membrane of bacterial cells. 

In this work, we have addressed these limitations by direct conjugation of a single TEGylated Europium chelate (BHHTEGST) [29] to a DNA sequence specific to the intracellular ribosomal 16S rRNA of *S. aureus* [34], for use in a homogeneous luminescence in situ hybridisation (LISH) detection. The BHHTEGST–DNA probe was soluble in aqueous solution and stable throughout experimentation. This single TEGylated Europium–DNA probe labelled *S. aureus* to produce bright orange-red (Eu^3+^ 615nm), high-contrast TGL images with high SNR. The robustness of the single Europium chelate conjugated DNA probe was confirmed by the substantially brighter luminescence of *S. aureus* cells compared to the closely related species, *S. epidermidis* (negative control). *S. aureus* and *S. epidermidis* look alike under a microscope and are frequently detected together in the culture of human blood samples *S. aureus*.

Since our first reports of TGL microscopy were published [27,35], we have made many advances that have led to the development of relatively low-cost instrument modules [36,37]. For the present experiments, we used the gated autosynchronous luminescence detector (GALD) that can be fitted to a standard inverted epifluorescence microscope. This setup was used to obtain high-contrast luminescent images of *S. aureus* cells, which were observed to have a SNR nearly 8-fold higher than that seen in the *S. epidermidis* cells.

## 2. Results and Discussion

Herein we describe the application of a single TEGylated Europium chelate for direct conjugation to short DNA specific to the ribosomal 16S rRNA of *S. aureus* for selective and highly sensitive detection of *S. aureus* via luminescent in situ hybridisation (LISH).

This luminescent BHHTEGST–Eu^3+^–DNA probe is soluble in water, stable and was successfully hybridised to intracellular rRNA of *S. aureus* to produce bright and high-contrast time-gated luminescence (TGL) images. This single Europium-conjugated DNA probe provides simplicity of preparation and improvement in intracellular targeting via in situ hybridisation over existing BSA/streptavidin/biotinylated DNA platforms.

### 2.1. Preparation of Europium Chelate Probes

BHHTEGST has a succinimide reactive group that was covalently bound to the modified DNA containing a primary amino group at 5′ position using NaHCO_3_ buffer (100 mM, pH 8.5) for incubation for 1 hr at 37 °C. The Europium chelate conjugated to DNA (DNA–BHHTEGST–Eu^3+^) described in this work was purified by precipitation of conjugated oligonucleotide in ethanol. We used 20-molar excess of Europium chelate to DNA for the conjugation, and only conjugated oligonucleotide was precipitated and isolated from excess unbound Europium chelate in ethanol solution (as detailed in the experimental section). We also observed that the initial purification of the unconjugated amino modified DNA by precipitation in ethanol was necessary for quantitative conjugation of DNA to the Europium chelate.

**Figure 1:**
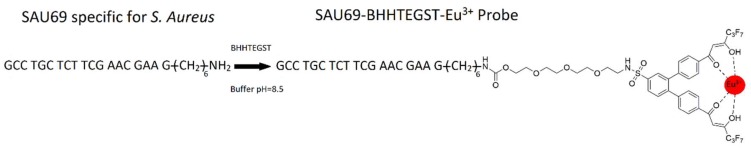
The SAU69 oligonucleotide that has a specificity for hybridisation to the 16S ribosomal RNA of *S. aureus* (with an amino group in 5′) was conjugated with Europium chelate (BHHTEGST–Eu^3+^) to produce the SAU69-BHHTEGST–Eu^3+^ probe.

Target DNA (SAU69; 5′-G AAG CAA GCT TCT CGT CCG), specific to the ribosomal 16S rRNA of *S. aureus* [34] was conjugated to the Europium chelate (BHHTEGST). This produced SAU69–BHHTEGST, as shown in Figure 1. The DNA–BHHTEGST probes were found to be soluble in aqueous solution and stable throughout experimentation for over one year. The universal nonselective eubacteria probe DNA sequence (EUB338; 5′-G CTG CCT CCC GTA GGA GT) [38] was used as a positive control. It labels both *S. aureus* and *S. epidermidis* bacteria. This probe was applied using the same treatment as that used when the SAU69 LISH was applied. The nonbinding NON-EUB338 probe (NON-EUB338; 5′-C GAC GGA GGG CAT CCT CA), reported by Wallner et al. [39] was used as a negative control. This sequence does not bind to most bacterial ribosomal 16S RNA. 

After purification of DNA–BHHTEGST by precipitation, a small portion of the conjugated probes was analysed by gel electrophoresis and analytical HPLC. The results from both tests indicated that the conjugation of Europium chelate BHHTEGST with DNA proceeded quantitatively without the presence of unreacted DNAs and so did not require further purification. The results from the HPLC analysis indicated that the conjugated DNA–BHHTEGST were more than 97% pure. This was confirmed by gel electrophoresis analysis (as described next). The prepared DNA–BHHTEGST conjugate was stored in a freezer (at −20 °C) as the stock probe solution (1.4 mM in concentration). 

**Figure 2:**
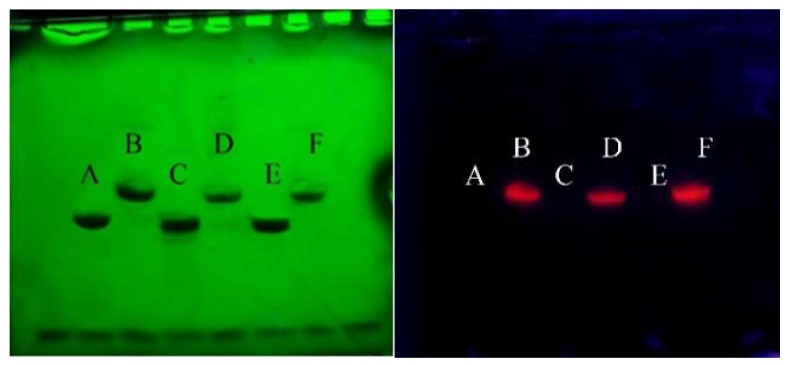
Gel electrophoresis results for EUB338/NON-EUB338/SAU69 DNAs and the corresponding DNA–BHHTEGST–Eu^3+^ conjugates (A, EUB338–DNA; B, EUB338–BHHTEGST–Eu^3+^; C, NON-EUB338–DNA; D, NON-EUB338–BHHTEGST–Eu^3+^; E, SAU69–DNA; F, SAU69-BHHTEGST–Eu^3+^) are shown. The images in the left-hand panel were illuminated with 254 nm (both unconjugated and conjugated DNAs absorb at this wavelength). Those in the right-hand panel were illuminated with 365 nm (only the DNA–BHHTEGST–Eu^3+^ conjugates emit red Europium luminescence at 613 nm).

Gel electrophoresis was performed to confirm the purity of the conjugated oligonucleotides (DNA–BHHTEGST). Unconjugated DNA was used as a control. Figure 2 (left-hand panel) shows the DNAs (EUB338, NON-EUB338 and SAU69) and their corresponding DNA–BHHTEGST–Eu^3+^ conjugates. Lanes A, C and E are unconjugated DNA tested (A, EUB338; C, NON-EUB338; E, SAU69). Lanes B, D and F are the corresponding DNA–BHHTEGST–Eu^3+^ conjugates (B, EUB338–BHHTEGST–Eu^3+^; D, NON-EUB338–BHHTEGST–Eu^3+^; F, SAU69–BHHTEGST–Eu^3+^). On the left-hand panel in Figure 2, DNA traces were visualised under 254 nm UV light. These include unconjugated DNAs (A, C and E) and their corresponding BHHTEGST conjugates (B, D and E). No unconjugated DNAs (A, C and E) were detected in B, D and F, confirming quantitative conjugation of DNA to BHHTEGST. For the visualisation of the Europium chelate in conjugated oligonucleotides, the same gel was sprayed with a solution of EuCl_3_. It was then visualised under 365 nm UV light. This is shown in the right-hand panel in Figure 2. The red Europium (III) emissions of B, D and F, seen as single spots under 365 nm radiation, confirmed a single pure product from conjugation of BHHTEGST–Eu^3+^ to each DNA oligonucleotide. The results confirmed the high purity of the conjugated oligonucleotides.

**Figure 3:**
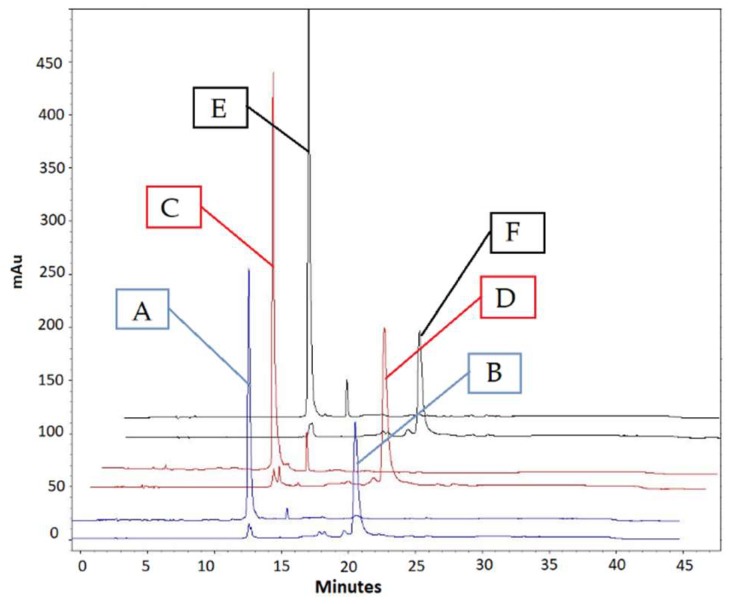
HPLC chromatograms of the free DNAs (A, EUB338; C, NON-EUB338; E, SAU69) and the corresponding DNA–BHHTEGST conjugates (B, EUB338–BHHTEGST; D, NON–EUB338-BHHTEGST; F, SAU69–BHHTEGST) are shown. Chromatograms were obtained using 270 nm excitation. (Please refer to SI for chromatograms of DNA–BHHTEGST at 270 nm and 330 nm).

Conjugated oligonucleotides were also analysed by analytical HPLC, and the purity of conjugated DNAs was confirmed according to HPLC chromatograms. As shown in Figure 3, chromatograms A, C and E represent unconjugated DNAs (A, EUB338; C, NON-EUB338; E, SAU69) and the chromatograms of B, D and F correspond to DNA–BHHTEGST conjugates (B, EUB338–BHHTEGST; D, NON-EUB338–BHHTEGST; and F, SAU69–BHHTEGST). The HPLC chromatograms of the unconjugated DNAs and conjugated DNAs were obtained at 270 nm excitation. The HPLC chromatograms of conjugated DNAs (DNA–BHHTEGST) were obtained at both 270 (Figure 3) and 330 nm (Figures S1–S9) excitation. DNA absorbs light at 270 nm and the chromophore present in BHHTEGST chelate at 330 nm wavelength (λ_max_ 335 nm). DNA–BHHTEGST absorption at both 270 nm and 330 nm confirmed that chelate was attached to the corresponding DNAs. Trace amounts of unidentified impurities were observed in the chromatogram of free DNAs. These impurities were minimal based on the relative integration value of the chromatograms. Trace impurities were also observed in the HPLC chromatogram of the conjugated DNAs. However, the amount was also negligible. According to the integration calculations obtained from the chromatogram, the average purity of the DNA–BHHTEGST conjugates was above 97%. Note that the Europium (III) ion was not added to the DNA conjugate for HPLC analysis. Through this manuscript, DNA–BHHTEGST refers to conjugated DNA with Europium ligand without addition of Europium (III) ion, and DNA–BHHTEGST–Eu^3+^ refers to the conjugated DNA with Europium chelate that binds the Europium (III) ion. 

The purity of the DNA–BHHTEGST probes played a crucial role for the TGL LISH imaging of *S. aureus*. Unconjugated Europium chelates are small molecules and so tend to bind nonspecifically to the cell membrane and subcellular entities. This creates a highly luminescent unwanted background signal. To counter this, the DNA–BHHTEGS probes were prepared as described to a high purity before their use.

Photophysical properties of DNA–BHHTEGST–Eu^3+^ (DNA = SAU69, EUB338 and NON-EUB338), including UV-visible absorption and luminescence emission spectra, were also investigated. UV-visible absorption analysis of the conjugated oligonucleotides indicated two maximum UV absorbance at 335 nm (for the Europium chelate) and at 260 nm (for the DNA), confirming the conjugation of Europium chelate to the oligonucleotides and validating the gel electrophoresis and HPLC data (Figure 2 and Figure 3). The luminescence spectra of conjugated oligonucleotides (DNA–BHHTEGST–Eu^3+^) emit luminesce light at 613 nm when excited at 335 nm (See Figures S10 and S11).

As shown in Supplementary Figures S10 and S11, for each conjugated DNA, luminescence intensity in milliQ water and Fluorescence Enhancing Buffer [40] (FEB) was measured using a gate delay of 100 µs on an Agilent Cary Eclipse fluorescence spectrophotometer. For each compound, luminescent intensity was lowest in water and became enhanced in a FEB solution. The FEB contained Trioctylphosphine oxide, where the phosphine oxide group provided a coordination bond to the Europium (III) metal ion and the trioctyl hydrophobic chain repelled the water molecules. This resulted in a reduction of the quenching effect of the water molecule on the Europium (III) metal ions in its excited state and consequently increased the luminescent intensity of the Europium chelate. Because of the increase seen in luminescence output, FEB was added to the stained cells for all the results reported here. 

### 2.2. LISH Protocol Optimisation and TGL Imaging

**Figure 4:**
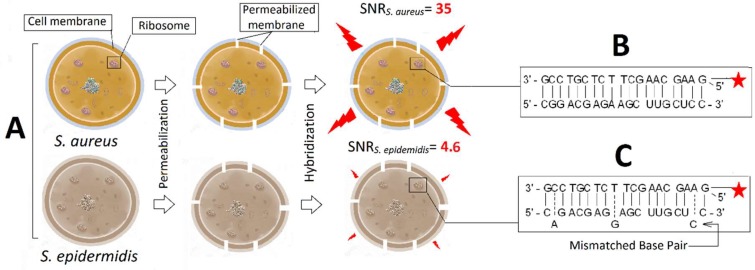
(**A**) Both *S. aureus* and *S. epidermidis* were fixed and permeabilised in parallel and then in situ hybridised with the SAU69–BHHTEGST–Eu^3+^ probe. (**B**) The 19-mer SAU69 probe aligns with *S. aureus* 16S rRNA at the 69 position and (**C**) has three mismatches to the *S. epidermidis* rRNA (mismatches are shown in the offset). A SNR of 35 was obtained for the TGL images of *S. aureus* versus a signal-to-noise ratio (SNR) of 4.6 for *S. epidermidis*. The Europium (III) ion was added after hybridisation with 4,6-Diamidino-2-phenylindole dihydrochloride hydrate (DAPI) for TGL imaging.

The work flow of homogeneous luminescent in situ hybridisation (LISH) labelling of *S. aureus* and *S. epidermidis* (negative control) with DNA–BHHTEGST probe is shown in Figure 4. The SAU69 DNA specific for *S. aureus* with amine modification at 5′ was conjugated quantitatively to the NHS moiety on the TEGylated Europium chelate (BHHTEGST) (Figure 1). *S. aureus* and *S. epidermidis* cells were fixed, permeabilised (lysozyme solution) and hybridised to the SAU69 specific DNA–BHHTEGST–Eu^3+^ probe (Figure 4A). The DNA sequence of SAU69 (5′-G AAG CAA GCT TCT CGT CCG) is specific to the ribosomal 16S rRNA of *S. aureus* [34]. The SAU69 probe is not specific to the 16S rRNA sequence of *S. epidermidis* due to three mismatch base pairs [41,42]. These three mismatches were enough to discriminate between *S. aureus* and *S. epidermidis* on the basis of their label intensity [42]. The alignments of the SAU69 oligonucleotide probe to its target 16S rRNA in *S. aureus* and its nontarget 16S rRNA in *S. epidermidis* are shown in Figure 4B,C. This difference made it possible for *S. aureus* to be selectively hybridised and imaged by TGL microscopy at a high intensity, so that it was easily discriminated from the closely related *S. epidermidis*. High-contrast TGL images were obtained of *S. aureus* with a SNR nearly 8-fold higher than *S. epidermidis* (SNR_S. aureus_ = 35, SNR_S. epidermidis_ = 4.6).

As described in the Experimental section (LISH procedure), the bacterial suspension was fixed before it was permeabilised in situ on the microscope slides. The hybridisation buffer containing the DNA probes solution [DNA–BHHTEGST; DNA–EUB338, NON-EUB338 and SAU69] was added to each well containing these fixed cells and incubated for 2 h. The slides were washed with a wash buffer (WB) to remove any unhybridised DNA probe and the LISH analysis buffer (containing FEB, DAPI and EuCl_3_) was added before visualising the cells with a time-gated microscope.

Optimal hybridisation conditions were determined by (i) varying the hybridisation parameters while analysing the (ii) TGL images of the stained *S. aureus* (acting as the target pathogen) and *S. epidermidis* cells (negative control). The mean intensity of the cell target signal (S) and the mean of the noncell signal (N) was then compared. For hybridisation, the buffer was prepared and tested at various concentrations of formamide (5 to 40%) and NaCl (0.1 to 1.0 M). According to Poppert et al. [42], optimal formamide concentration is dependent on the particular oligonucleotide sequence tested. The NaCl concentration in the washing buffer depended on the formamide concentration used in the hybridisation buffer. 

The hybridisation buffer resulting in the strongest signal was observed to contain 10% (*w*/*v*) formamide, 0.9 M NaCl, 20 mM Tris-HCl pH 8.0, and 0.01% sodium dodecyl sulfate (SDS) dissolved in milliQ water. The optimised hybridisation temperature and time were optimised at 70 °C and 10 min. To minimise nonspecific binding, the samples were then cooled to 46 °C (over 30 min). Next, samples were incubated at 46 °C for a further 2 h. The washing buffer was optimised at 0.46 M NaCl, 20 mM Tris-HCl pH 8.0, and 0.01% SDS. The washing buffer was preheated to 46 °C before incubating it with the samples for 5 min. Slides were flushed with milliQ water, dried with a gentle air blast and then loaded with 2 µL of LISH analysis buffer containing the FEB buffer, DAPI and EuCl_3_. After 15 min incubation, the slides were visualised with the time-gated microscope described above. 

### 2.3. Differentiation between S. aureus and S. epidermidis

**Figure 5:**
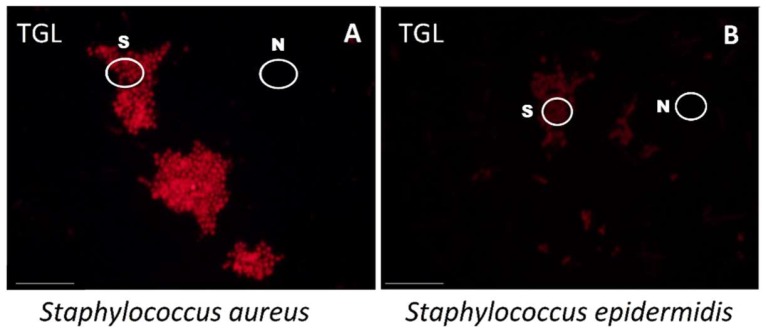
(**A**) *S. aureus* cells labelled with the SAU69–BHHTEGST–Eu^3+^ LISH probe illuminated in the TGL mode are shown. (**B**) *S. epidermidis* cells labelled with SAU69–BHHTEGST–Eu^3+^ collected under identical conditions are shown. The scale bar indicates 10 µm. The circles added indicate the areas selected to calculate the mean signal intensity of the desired target cell (S) and the undesired noise (N).

*S. aureus* was able to be selectively hybridised and imaged by TGL microscopy at a high intensity, so that it was easily discriminated from the closely-related *S. epidermidis*. The conjugated DNA (SAU69–BHHTEGST), indicated by the intensity of its signal, bound more to the *S. aureus* than to the *S. epidermidis* when prepared and tested in different wells on the same slide. As shown in Figure 5, (A) *S. aureus* cells were observed to be labelled with much higher signal intensity in comparison with (B) *S. epidermidis* cells using the SAU69 probe specific for *S. aureus*. The circles added to indicate the areas selected randomly to quantify the mean-signal-intensity of the desired target cells (S) and the background noise (N). 

**Figure 6:**
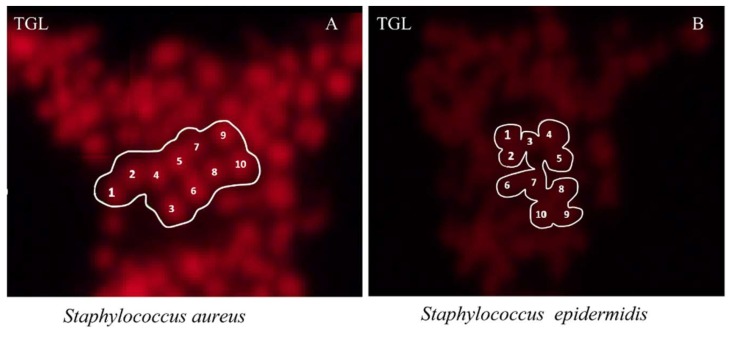
(**A**) The circles added indicate the 10 cells of (**A**) *S. aureus* and (**B**) *S. epidermidis* randomly selected for calculating an average SNR. (**A**) The mean signal of the 10 cells of *S. aureus* was calculated at S = 95.7, N = 3.1 and SNR = 31. (**B**) *S. epidermidis* was calculated at S = 32, N = 6.9 and SNR = 4.6.

As shown in Figure 6, in each circle, 10 target cells were selected randomly for the quantification of their signal (S). The SNR of the *S. aureus* (SNR = 31) was nearly 8-fold higher than that of the *S. epidermidis* (SNR = 4.6) images (see Supplementary Figures S12–S17). The EUB338 staining positive control confirmed that the reduced SAU69 signal seen in the *S. epidermidis* cells was not caused by poor probe penetration, but by the target mismatch of the SAU69 probe. 

To support this observation, a commercially available fluorescent probe (SAU69-Alexa Fluor 488, excitation at 488 nm) was also used as a positive control for comparison staining of the *S. aureus* cells. The hybridisation was then optimised to maximise fluorescence [21,22,23] and luminescence yield at the lowest background emission for comparison with the LISH labelling with the SAU69 specific DNA–BHHTEGST–Eu^3+^ probe. After obtaining the corresponding images, the detection sensitivity or SNR of the luminescent Europium probe (SAU69–BHHTEGST–Eu3+) in comparison with fluorescent probe (SAU69-Alexa Fluor 488) for the staining of *S. aureus* was investigated. ImageJ software was used to quantify the intensity of pixels in the raw images collected of cells stained with both probes. The average SNR calculated for the Europium staining of *S. aureus* cells was 6-fold higher than that calculated for the Alexa Fluor 488 stained cells. This confirms that time-gated luminescence (TGL) imaging provides higher sensitivity (SNR) of detection by eliminating the autofluorescence background compared to existing conventional fluorescence probes (Figures S18 and S19). 

### 2.4. Positive/Negative Controls

**Figure 7:**
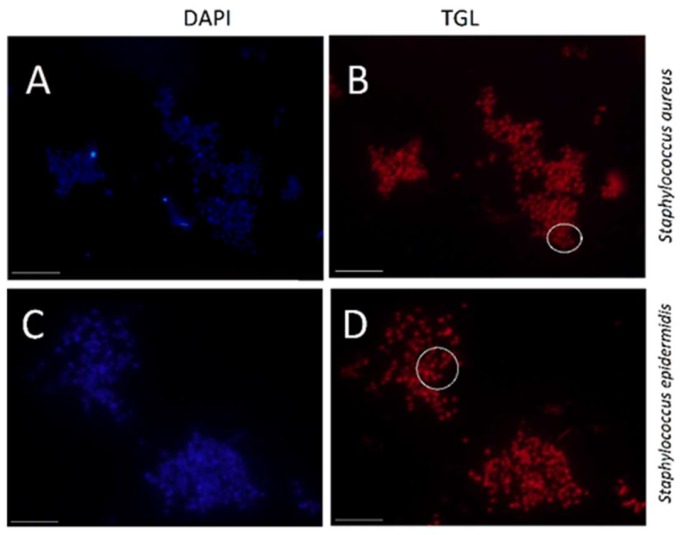
Images of both *S. aureus* (**A**,**B**) and *S. epidermidis* (**C**,**D**) stained with the EUB338–BHHTEGST–Eu^3+^ probe are shown. (**A**,**C**) DAPI nuclear staining; (**B**,**D**) time-gated luminescence (TGL) staining are shown. Oil immersion 100× objective and TGL exposure times at 2.0 Sec. were used. The scale bar indicates 10 μm. (**B**) *S. aureus* is at S = 72.1, N = 5 and SNR = 14.4. (**D**) *S. epidermidis* is at S = 81, N = 5 and SNR = 16.2.

The universal eubacteria probe, EUB 338 conjugated to BHHTEGST, was applied to *S. aureus* and *S. epidermidis* and used as a positive control in these tests. Both *S. aureus* and *S. epidermidis* cells were treated identically to those stained with the SAU69 probe using different wells on the same slide. The EUB338 complementary 16S RNA sequence is common to most bacteria [38], and *S. aureus* and *S. epidermidis* were stained by the EUB338–BHHTEGST–Eu^3+^ probe almost equally (Figure 7). The stain had a mean pixel value of 72.1 and 81 for *S. aureus* and *S. epidermidis* cells, respectively (Figures S14 and S15).

**Figure 8:**
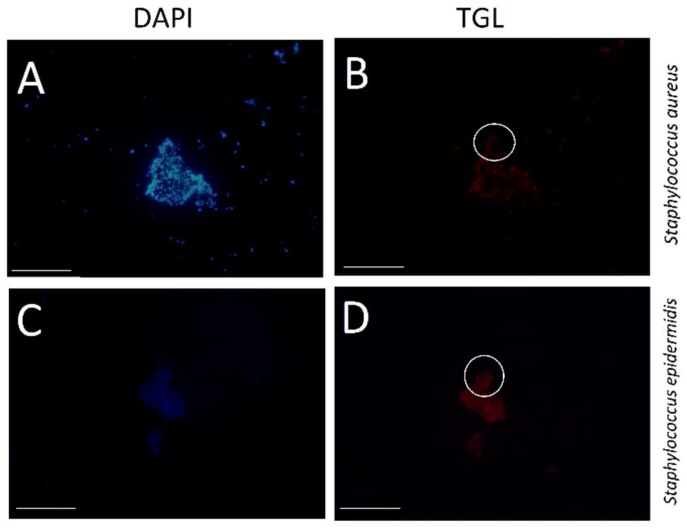
Images of both *S. aureus* (**A**,**B**) and *S. epidermidis* (**C**,**D**) stained with the NON-EUB338–BHHTEGST–Eu^3+^ probe are shown. (**A**,**C**) DAPI nuclear staining; (**B**,**D**) time-gated luminescence (TGL) are given. Oil immersion 100× objective and TGL exposure times (2.0 Sec.) were used; the scale bar indicates 10 μm. (**B**) *S. aureus* at S = 36.7, N = 6.1 and SNR = 6 and (**D**) *S. epidermidis* at S = 33.8, N = 7.3 and SNR = 4.6.

The NON-EUB338 probe described by Wallner et al. [39] was used as a negative control. Its sequence is absent in most bacterial RNA. The NON-EUB338–BHHTEGST–Eu^3+^ probe had limited staining, with a mean pixel value of 36.7 and 33.8 for *S. aureus* and *S. epidermidis*, respectively (Figure 8 and Supplementary Figures S16 and S17). These control experiments confirmed that the in situ hybridisation technique was highly specific.

**Figure 9:**
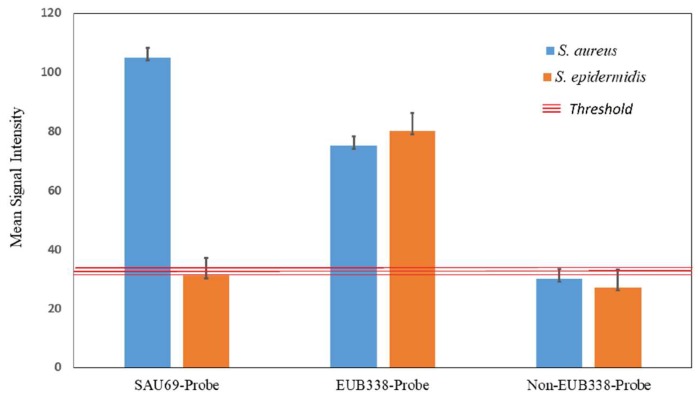
The *S. aureus* and *S. epidermidis* labelled with SAU69–BHHTEGST–Eu^3+^, EUB338–BHHTEGST–Eu^3+^ and NON-EUB338–BHHTEGST–Eu^3+^, the threshold of the luminescence intensity and standard deviation (SDV) are shown as a mean (n replication = 30) signal intensity (See SI Table S2).

Figure 9 shows the averaged results (*n* = 30) of luminescent stained *S. aureus* and *S. epidermidis* visualised by LISH. The mean signal intensity for *S. aureus* was 3.5 times higher than that for *S. epidermidis* for the SAU69–BHHTEGST–Eu^3+^ probe. Further, for the same probe (SAU69–BHHTEGST–Eu^3+^), the mean signal-to-noise ratio (SNR) of *S. aureus* was 8 times higher than that for *S. epidermidis* (Supplementary Figures S12 and S13, Table S2). 

The EUB338–BHHTEGST–Eu^3+^ (positive control) probe labelled both *S. aureus* and *S. epidermidis* with a similar intensity. Luminescent intensity of the images in the negative controls (NON–EUB338) was negligible, which suggests the presence of some minor nonspecific binding of probes within the cells. However, the mean luminescent intensity of the images with the positive probes (SAU69 and EUB338) was consistently much higher than the threshold of the negative controls (NON-EUB338), as depicted in Figure 9. Specifically, labelled *S. aureus* (SAU69–BHHTEGST–Eu^3+^) was 3.2 times brighter than the same cells labelled with the NON-EUB338–BHHTEGST–Eu^3+^ probe (negative control).

It is a common approach to classify the fluorescence in situ hybridisation (FISH) images into target (positive) cells and nontarget (negative) cells based on a threshold of the fluorescence intensity or signal-to-noise ratio (SNR) [43,44,45,46,47]. It means all cells with higher fluorescence intensity than a certain threshold are classified as target cells. Those cells with a signal less than this threshold were classified as nontarget cells. For example, Pernthaler et al. [44] developed automated digital microscopy to facilitate the processing of a large number of FISH-stained samples based on a threshold level classification that was determined on samples hybridised with the NON-EUB338 probe as negative control. The positive FISH-stained targets were classified based upon their higher signal intensity or higher signal-to-noise ratio relative to the threshold of the signal from the negative control. 

This simple threshold-based classification approach used with our data was confirmed with the mean luminescent signal intensity of the positive control probes observed to be consistently higher than the threshold of the mean signal from the negative probes. Specifically, both EUB338 labelled *S. aureus* and *S. epidermidis* showed the same mean signal intensity of ~3.2 times higher signal intensity than the threshold of signal from the negative control (NON-EUB338). Using this negative control threshold of NON-EUB338 probe staining (as shown in Figure 9), the SAU69–BHHTEGST–Eu^3+^ probe specifically stained *S. aureus* with a mean signal intensity of 3.5 times higher than the threshold level of signal from *S. epidermidis*. 

## 3. Conclusions

In summary, homogeneous luminescent in situ hybridisation (LISH) assays with time-gated microscopy provide inexpensive, sensitive and rapid identification of pathogenic bacteria. Homogeneous fluorescent in situ hybridisation (FISH) has been extensively used for a wide range of applications in comparison with other molecular methods such as PCR, nanoparticle DNA-based biosensor and DNA microarray approaches, which have their own specific and limited applications. This report provides a proof-of-principle for LISH detection of *S. aureus* with high specificity and sensitivity (SNR) and its differentiation from a close relative *S. epidermidis*. LISH compared to FISH has the advantage of eliminating the background autofluorescence, which is a major limitation of conventional fluorescence microscopic assays. LISH compared to the conventional blood test detection method can be performed at an at least 10-fold less cost in term of time taken.

The direct conjugation of a single Europium chelate to a DNA probe provides ease and improvement over existing BSA/streptavidin/biotinylated DNA, and more importantly, the smaller size of the conjugate facilitates hybridisation through permeabilised bacterial cell membrane to target intracellular rRNA. We believe the current study provides an effective alternate sensitive protocol for the detection of intracellular DNA/RNA targets by luminescent in situ hybridisation.

## 4. Experimental Section

### 4.1. Materials

4,6-Diamidino-2-phenylindole dihydrochloride hydrate (DAPI) (D9642), Europium (III) chloride hexahydrate (203254), paraformaldehyde (P6148), sodium tetraborate decahydrate (1303-96-4), sodium acetate (S3272), lyophilised powder (lysozyme from chicken egg white; L6876), tris(hydroxymethyl)aminomethane (tris base -252859), boric acid (B6768), ethylenediaminetetraacetic acid (EDTA, E9884), formamide (47670), sodium dodecyl sulfate (SDS, L3771), N,N-dimethylformamide (DMF, 227056), dimethyl sulfoxide (DMSO, 472301), tetramethylethylenediamine (TEMED-T7024), sodium persulfate (216232), 30% acrylamide (contains 1% methylene bis acrylamide) (A3574), urea (U5378) and agarose (A0169) were purchased from Sigma-Aldrich, Australia and used without further modification. DNA oligonucleotides modified at their 5′-end with an amino group, including the sequences EUB338 (nonselective probe), NON-EUB338 (negative control probe) and SAU69 specific for *S. aureus*, were purchased from Integrated DNA Technologies Australia. *S. aureus* and *S. epidermidis* cells (from patient samples that were first identified via blood-culturing and PCR) were provided by Westmead Hospital, Sydney, Australia with permission. Teflon coated microscope slides (G401-12) containing wells (7 × 3) were purchased from ProSciTech, Australia.

### 4.2. The Conjugation of Europium Chelate to DNA

BHHTEGST, utilising a succinimide reactive group, was covalently attached to a modified DNA containing a primary amino group at its 5′-residue using NaHCO_3_ buffer (100 mM, pH of 8.5) for incubation for 1 hr at 37 °C. Purification was carried out using a common ethanol precipitation procedure. A total of 100 μL of milliQ water solution containing DNA (SAU69 [5′-amino-G AAG CAA GCT TCT CGT CCG] 1086.4 µg, 176.7 nmol) was prepared as a stock solution. Then, 10 μL aliquot of this was purified by precipitation by adding sodium acetate solution (2 μL, pH 5.5, 3M) and ethanol (400 μL). The mixture was vortexed and kept at −80 °C for 15 min. The precipitated DNA was then collected by centrifuging for 2 min at 16 K rpm. The precipitated DNA was dissolved in 10 μL of milliQ water. This procedure was repeated twice more to obtain pure DNA.

Pure DNA was dissolved in 10 μL of milliQ water. A total of 10 μL of sodium tetraborate (0.2 M, pH 8.5) was added. This was followed by the addition of 20 μL of BHHTEGST solution (10 mg in 500 μL of DMSO, 20 mM to form molar excess of BHHTEGST to DNA of 20). The mixture was incubated for one hour at room temperature to complete the conjugation of BHHTEGST to DNA. Excess BHHTEGST was removed from the formed conjugated oligonucleotide probe by its precipitation in ethanol. A total of 2 μL of sodium acetate solution (pH 5.5, 3M) and 400 μL of ethanol was added to this reaction mixture. The precipitates were collected by first cooling to −80 °C, then centrifuged again at 16 K rpm for 15 min. The procedure was repeated 3 times to obtain the crude probe extract SAU69–BHHTEGST. The same procedure was used to prepare the other DNA conjugates of EUB338–BHHTEGST and NON-EUB338–BHHTEGST. The purity of these conjugates was measured using gel electrophoresis and analytical HPLC techniques. The results are shown in Figure 2 and Figure 3 and in the SI. The DNA sequences used for in the conjugation are listed next:

EUB338: 5′-G CTG CCT CCC GTA GGA GT (acting as a nonselective probe);

NON-EUB338: 5′-C GAC GGA GGG CAT CCT CA (acting as a negative control probe);

SAU69: 5′-G AAG CAA GCT TCT CGT CCG (acting as a probe specific for *S. aureus*).

### 4.3. Electrophoresis Solution and Gel Preparation

Gel electrophoresis conductive medium 1X TBE solution was prepared by diluting 5X TBE solution (54 g Tris, 27.5 g boric acid and 500 mg EDTA) in 1 L of milliQ water. The gel was prepared with 20 mL of 30% acrylamide (containing 1% (w/v) methylene bis acrylamide), 12 g urea and 6 mL 5X TBE to 30 mL of milliQ water. Seven millilitres of this mixture was added to 30 μL of 10% sodium persulfate (at a concentration of 30 mg in 300 μL of milliQ water) and 3 μL of tetramethylethylenediamine (TEMED). This solution was quickly injected between two layers of glass. It was then held for 10 min until a gel formed.

### 4.4. Gel Imaging

After completion of the gel electrophoresis, both free and conjugated DNA with the Europium ligand were visualised by exciting with a UV-visible set at 254 nm. Crude precipitated DNA-BHHTEGST, after its conjugation and precipitation in ethanol, was dissolved in 10 μL of milliQ water, and 1 μL was then mixed with 5 μL of a marker (bromophenol blue (BPB) dissolved in 1X TBE) and applied to the electrophoresis pocket. After completing the gel electrophoresis, the gel was illuminated with a 260 nm UV lamp to identify the DNA and DNA probe. A few drops of EuCl_3_ solution (3 mM) were added to the gel, and the conjugated DNA–Europium chelate (DNA–BHHTEGST–Eu^3+^) was visualised under UV-visible light set to 365 nm. At this wavelength, only the DNA Europium conjugates were seen. The Europium moiety of DNA probe was excited by and emitted a red colour (613 nm) (Figure. 2).

### 4.5. Analytical HPLC Analysis of DNA Conjugates

For the analysis of DNA and conjugated DNA with Europium chelate, analytical reversed phase HPLC, DENALI C18 column (5 µm, 4.6 mm ID, 250 mm) at a flow rate of 1.0 mL/min, was used. A Shimadzu LC system consisting of a DGV-12A degasser, SIL-10AD auto-injector, and an SPD-M10A tuneable absorbance detector was coupled to the HPLC. Solvent system of solvent A (0.1 M triethylammonium acetate at pH 6.5) and solvent B (0.1 M triethylammonium acetate in 75% acetonitrile, pH 6.5) was applied at 10 to 100% B gradient concentrations over a 20-min period.

### 4.6. Photophysical Properties of DNA Conjugates

Photophysical properties of DNA–BHHTEGST–Eu^3+^ (DNA = SAU69, EUB338 and NON-EUB338), including UV-visible absorption spectra, were collected using a NanoDrop 2000 UV (Thermo Scientific) spectrometer. Luminescence data were captured with an Agilent Cary Eclipse Fluorescence Spectrophotometer. See SI for more details (Figures S10 and S11).

### 4.7. The Cells (S. aureus and S. epidermidis)

Blood agar plates of clinical isolates positive for *S. aureus* or *S. epidermidis* were collected from Westmead hospital (University Biosafety Committee approvals 09/14/LAB and 5201000927) after each isolate’s identity was confirmed using their own in-house polymerase chain reaction (PCR) technique [48]. Cells were prepared for testing by first growing to log phase, isolation by centrifugation and then their re-suspension in ethanol. This treatment made the cells viable and helped to fix their cellular architecture. Treated this way, they could be stored long-term and could be handled safely when tested.

### 4.8. Buffers

Hybridisation buffer (HB) was prepared with NaCl (360 µL, 5M) and Tris/HCl (40 µL, pH 8.0, 1M), formamide (200 µL), milliQ water (1.40 mL) and SDS (10% (w/v), 2 µL) and then made to form a volume of 2 mL. Washing buffer (WB) was prepared with NaCl (4.60 mL, 5M), Tris/HCl (1 mL pH 8.0, 1M), SDS (10%, 50 µL) and milliQ water (48.49 mL) and made up to a volume of 50 mL. LISH analysis buffer (LAB) was prepared by mixing fluorescence enhancement buffer (FEB) [40] (1 mL, 1 X, 5 µL of DAPI solution (1.0 µg/mL) and a Europium (III) chloride solution (10 µL of 3 mM). 

Preparation of fluorescence enhancing buffer solution [10× (FEB)] was done in accordance with Arnaud et al. [40]. A 44 mL solution of 0.1 M sodium hydroxide was prepared, and the pH was adjusted to 4.7 with glacial acetic acid, then 1% by volume of Triton X-100 was added. Trioctylphosphine oxide (38 mg) was dissolved in ethanol (5 mL) and added to the sodium acetate solution (1.25 mL), and then 1 × FEB was used for the experiments.

Lysozyme solution was prepared by dissolving lysozyme (15 mg) in 0.1 M Tris/HCl pH 8.0 (1 mL) just before its use. A laboratory-built hybridisation oven, equipped with a sealed chamber, was used for all incubations. It could hold 3 regular slides at once. It maintained an atmospheric humidity of 100%. Its temperature was maintained at ±0.5 °C.

### 4.9. LISH Procedure

A dilute solution of agarose (0.02% *w*/*v*) was prepared in milliQ water. A total of 20 µL of this solution was then pipetted onto wells in a 7 × 3 Teflon well coated slide (ProSciTech G401-12). The addition of agarose to the slides aided sample adhesion and appeared to improve fluorescent emission intensity. Each prepared slide was placed on a hot plate and dried at 80 °C for 10 min. The slides were then cooled to RT, and a bacterial suspension (10 µL) was added to each well at a concentration of around 1 × 10^6^ cells/mL. It was then dried briefly for a few minutes using a hot plate set to 30 °C. It is important for the slides not to be heated for too long or be overheated. Doing so causes damage to the cells. To ensure control over positive and negative labelling, the first row in each slide was loaded with *S. aureus* and the second with *S. epidermidis*.

For permeabilisation, 20 µL of lysozyme solution was added to each well. Slides were then incubated in 100% humid chamber at 37 °C for 1 h. Subsequently, slides were removed and gently washed with 3 × 100 µL aliquots of 95 % ethanol. 

From the probe stock solution [SAU69-BHHTEGST (100 µL, 1.4 mM)], 1 µL was added to 100 µL hybridisation buffer [14.1 pM probe/µL of hybridisation buffer (HB)], and then 10 µL of this was added to each well. The hybridisation oven was preheated to 70 °C. After adding 10 µL hybridisation buffer (HB) (with probe) to each well, the slides were loaded into the oven set to 100% atmospheric humidity. The slides were left at 70 °C for 10 min before cooling slowly to 46 °C, over a 30-min period. They were then held for another 2 h at 46 °C. After this, the slides were removed and each well washed with washing buffer (WB) 2 × 100 µL gently. Subsequently, they were immersed in 40 mL of pre-heated 46 °C wash buffer (WB) for 5 min.

After washing, the slides were flushed with milliQ water and dried using a gentle air blast. The wells were then loaded with 2 µL of LISH analysis buffer [FEB buffer (1 mL, 1X), 5 µL of DAPI solution (1.0 µg/mL) and the Europium chloride solution (10 µL of 3 mM)]. They were covered with a long-length glass cover-slip. After leaving the slides on the bench for 15 min to incubate, they were visualised with the time-gated microscope system described next. 

### 4.10. Imaging

The fixed and permeabilised *S. aureus* and *S. epidermidis* cells were hybridised and treated with LISH analysis buffer (described in the LISH procedure section of this work), and the thus labelled cells were then visualised using an Olympus CKX41 inverted microscope. EuCl_3_ solution was added with LISH analysis buffer after completing the labelling procedure to reduce nonspecific binding and aggregation of the DNA-probe. 

Bright-field, DAPI and time-gated luminescence (TGL) images were then captured with the Olympus CKX41 fluorescence microscope. Time-gated luminescence images were captured with the gated autosynchronous luminescence detector (GALD) system developed by Connally [37]. This was used by first inserting the unit into the differential interference contrast (DIC) slot on the microscope. For TGL Images, a DP72 colour camera set at an ASA speed of 400 and an exposure period of 2.0 s were used. Images were stored as TIFF files using the same UV lamp source and a DAPI filter, after an exposure of 10 ms DAPI. The images acquired were not further processed, enhanced or manipulated. 

The images of the *S. aureus* and *S. epidermidis* cells using the time-gated mode were captured with a DP72 Olympus camera. It was set at 400 ASA and an exposure of 2.0 s. Images were analysed with *ImageJ* software to calculate their signal-to-noise ratio (SNR). Images were acquired as a 24-bit (RGB) TIFF format image. The red histogram channel carried the image’s brightness information. The maximum brightness of an 8-bit pixel corresponded to a 255 value.

### 4.11. Quantification of Signal-to-Noise Ratio (SNR)

The mean signal intensity and signal-to-noise (SNR) of the stained cell images were quantified using *ImageJ* software (version 1.46r). *ImageJ* calculated the mean signal intensity (S) of an area within the target cell, and the noise (N) was calculated by comparing it to the mean pixel intensity of a randomly selected area containing no cells.

## Data Availability

The images and datasets generated and analysed during the current study are available from the corresponding author on reasonable request.

## Supplementary Materials

The Supporting Information is available on the *Molecules* Publications website at DOI: xxx. Analytical HPLC profile of the DNA conjugates and the quantification details of their signal-to-noise ratio (SNR) is provided here as well.
