# Supplementary material for: Time-Gated Luminescent In Situ Hybridization (LISH): Highly Sensitive Detection of Pathogenic Staphylococcus aureus
Source: Molecules. 2019 May 31;24(11):2083. doi: 10.3390/molecules24112083 (PMC6600140; doi:10.3390/molecules24112083)
Supplement: Supplementary file 1 [file molecules-24-02083-s001.pdf]

Supporting information:

Time-gated luminescent in-situ hybridization (LISH): highly sensitive detection of pathogenic Staphylococcus aureus

Nima Sayyadi,[a,b*] Russell Connally,[c] Thomas S. Lawson,[b,c] Jingli Yuan,[d] Nicolle H. Packer [a,b*] and James A. Piper [b,c]

a Department of Chemistry and Biomolecular Sciences, Macquarie University, Sydney. b ARC Centre of Excellence for Nanoscale Biophotonics (CNBP), Macquarie University, Sydney. c Department of Physics and Astronomy, Macquarie University, Sydney. d State Key Laboratory of Fine Chemicals, School of Chemistry, Dalian University of Technology, Dalian, China

Corresponding authors email: Nima.Sayyadi@mq.edu.au and Nicki.Packer@mq.edu.au

Contents Page

Analytical HPLC profile of conjugates S2-6
Quantification of signal, noise and signal-to-noise (SNR) ratio S7-10

HPLC data of conjugated oligonucleotides:

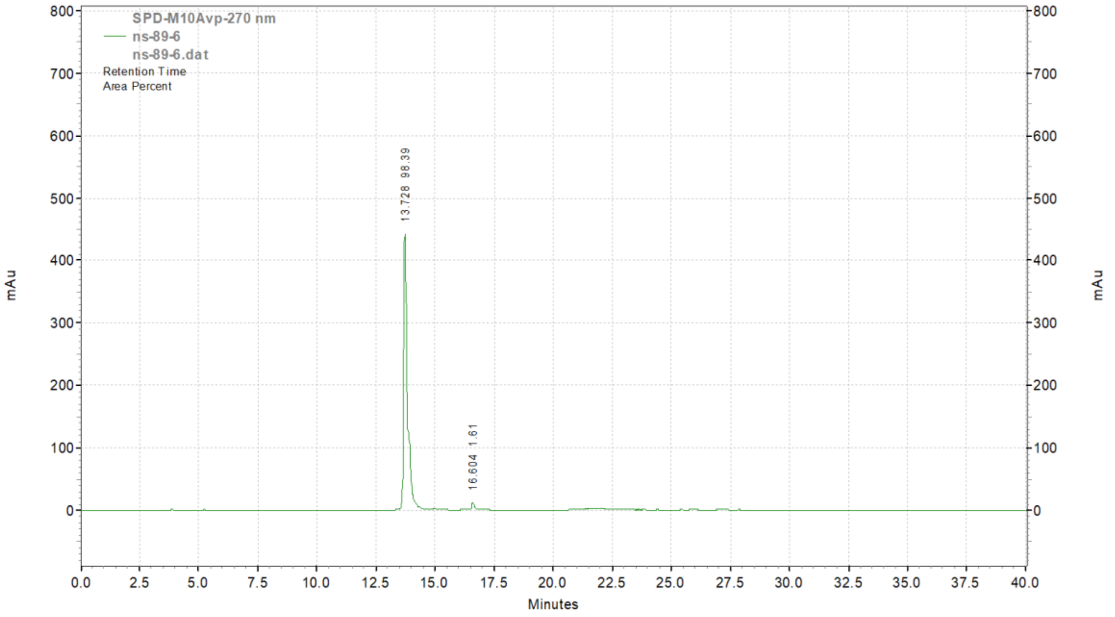

Figure S1: HPLC chromatogram of SAU69 DNA at 270 nm (Purity 98.4%, RT=13.7).

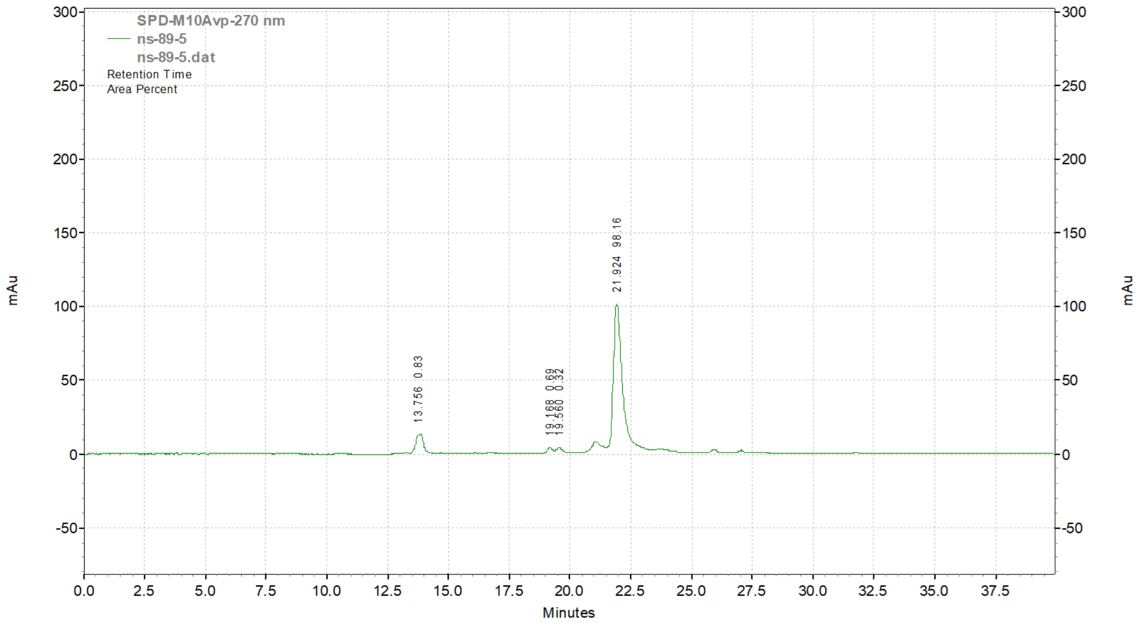

Figure S2: HPLC chromatogram of SAU69-BHHTEGT probe at 270 nm (Purity 98.1%, RT=21.9).

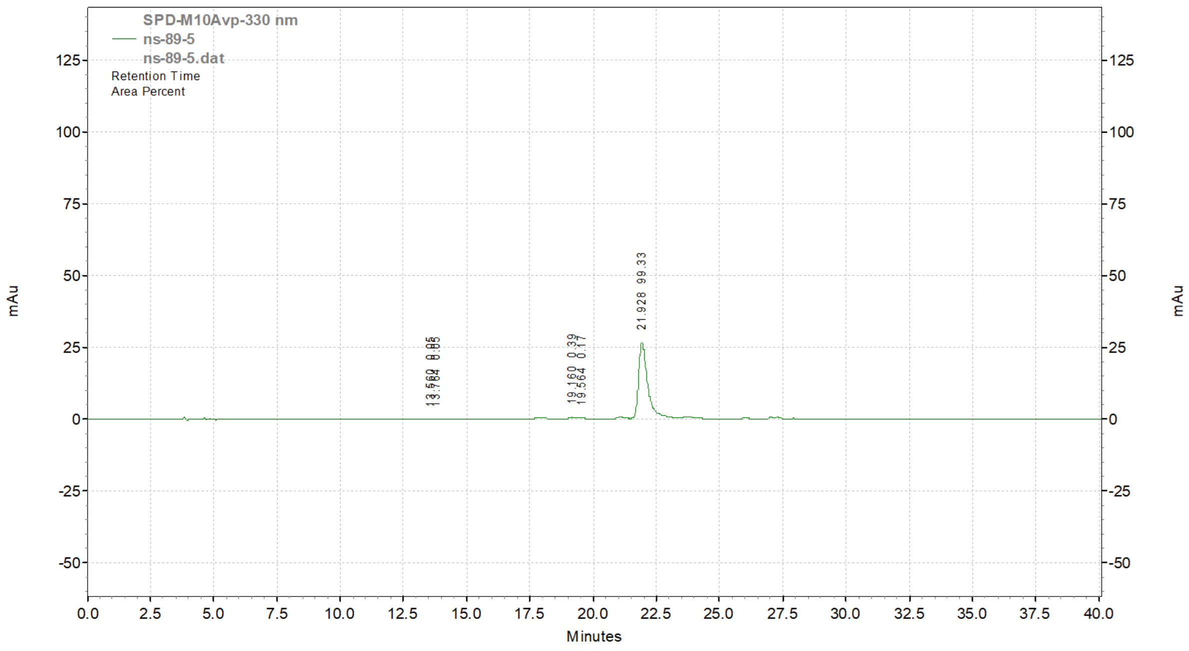

Figure S3: HPLC chromatogram of SAU69-BHHTEGST Probe at 330 nm (Purity 99.3%, RT=21.9).

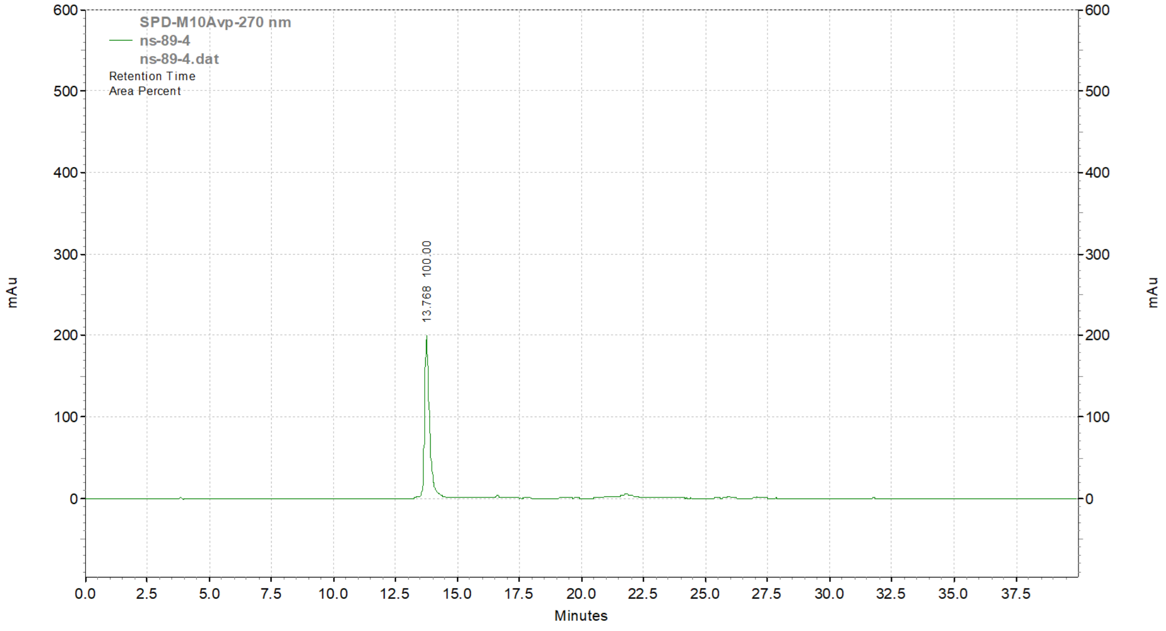

Figure S4: HPLC chromatogram of EUB338 DNA at 270 nm (Purity 100%, RT=13.77).

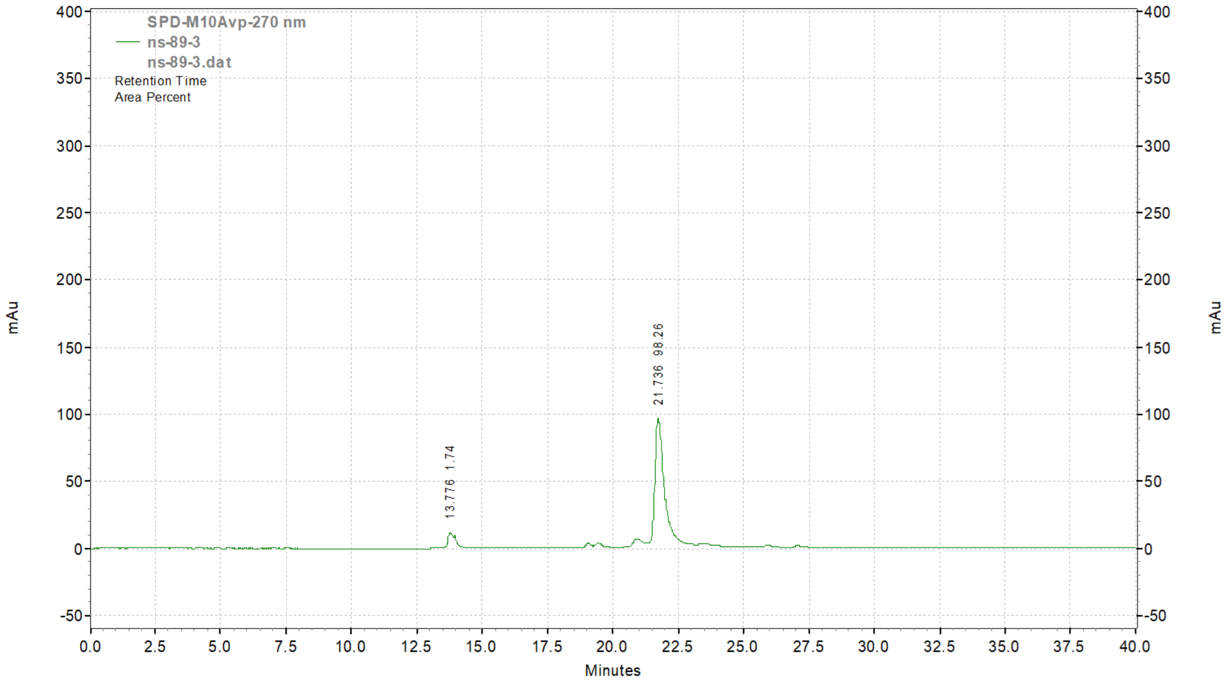

Figure S5: HPLC chromatogram of EUB338-BHHTEGST Probe at 270 nm (Purity 98.2%, RT=21.7).

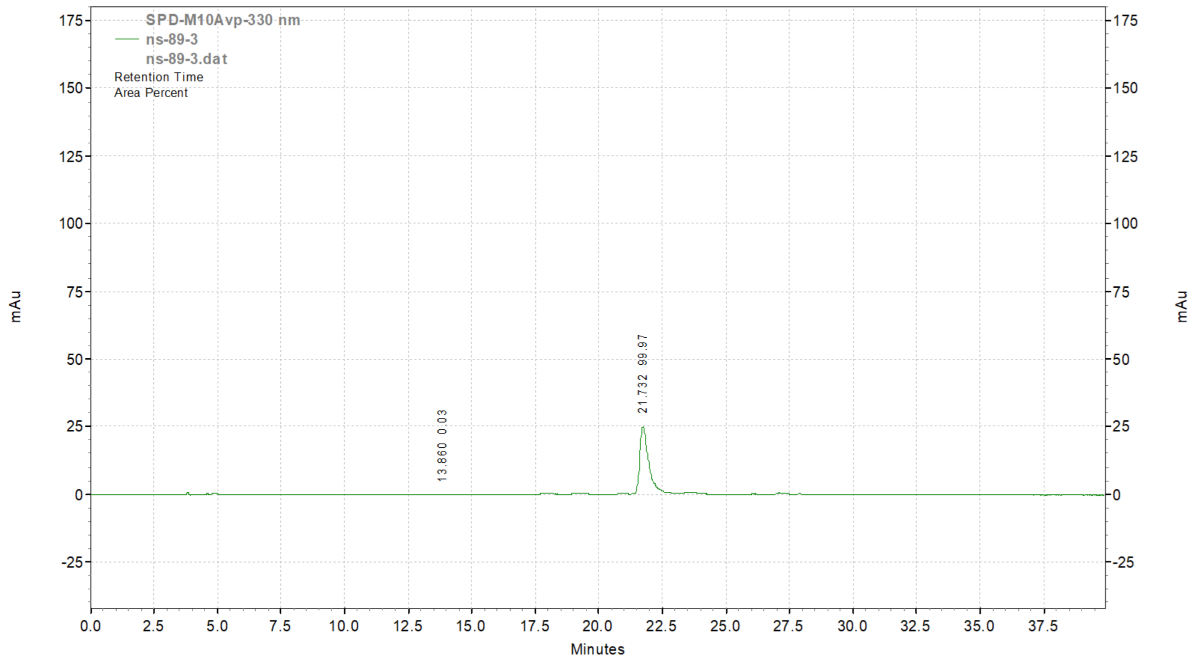

Figure S6: HPLC chromatogram of EUB338-BHHTEGT Probe at 330 nm (Purity 99.9%, RT=21.7).

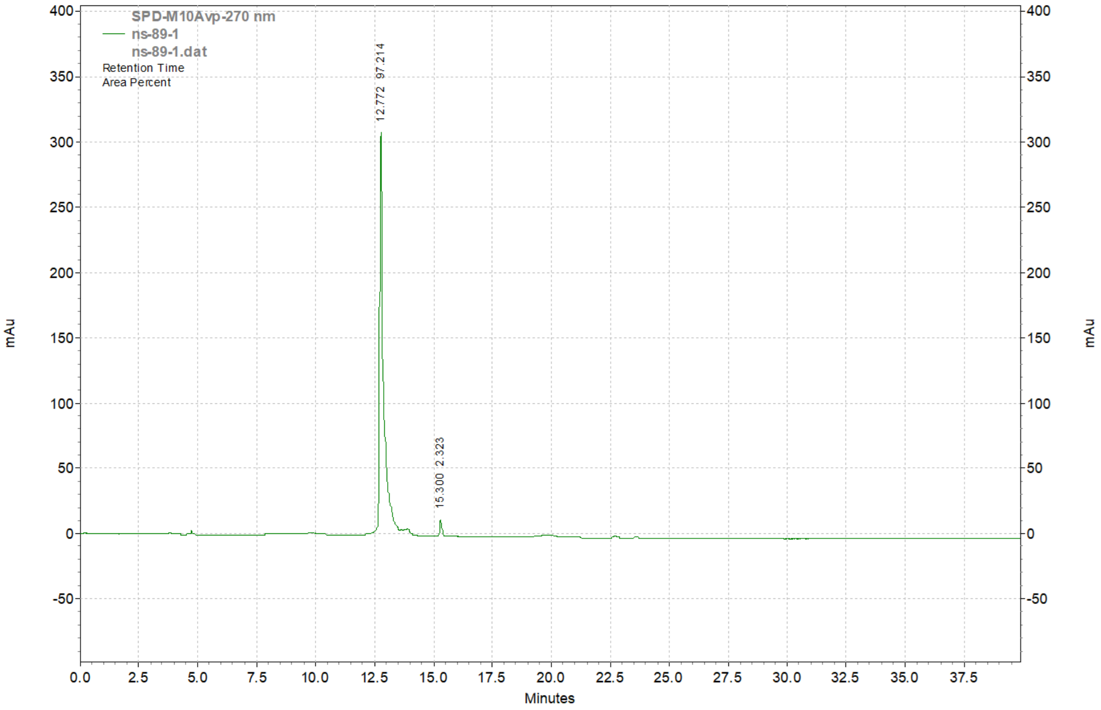

Figure S7: HPLC chromatogram of NON-EUB338 DNA at 270 nm (Purity 97.2%, RT=12.77).

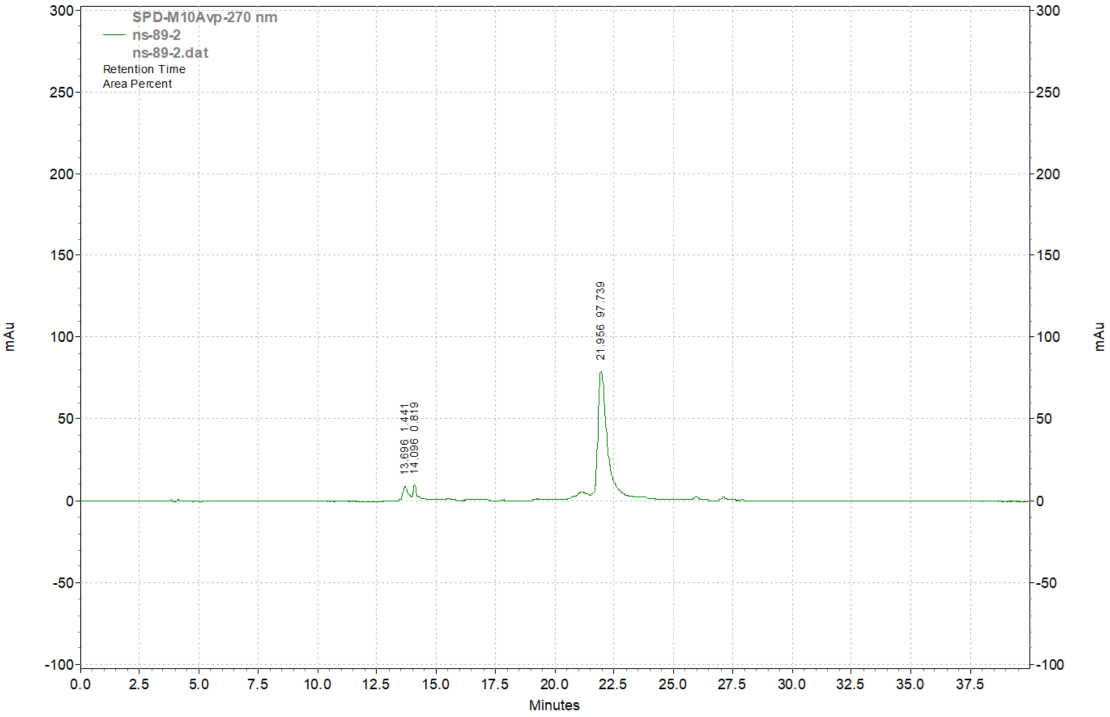

Figure S8: HPLC chromatogram of NON-EUB338-BHHTEGT Probe at 270 nm (Purity 97.7%, RT=21.96).

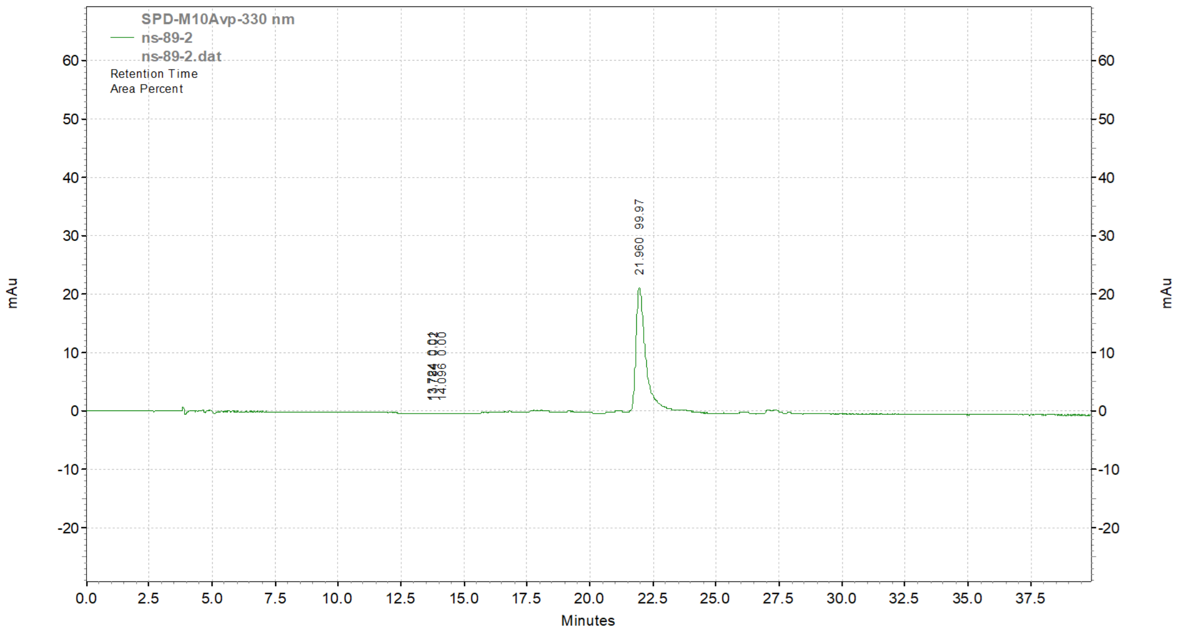

Figure S9: HPLC chromatogram of NON-EUB338-BHHTEGST Probe at 330 nm (Purity 99.97%, RT=21.96).

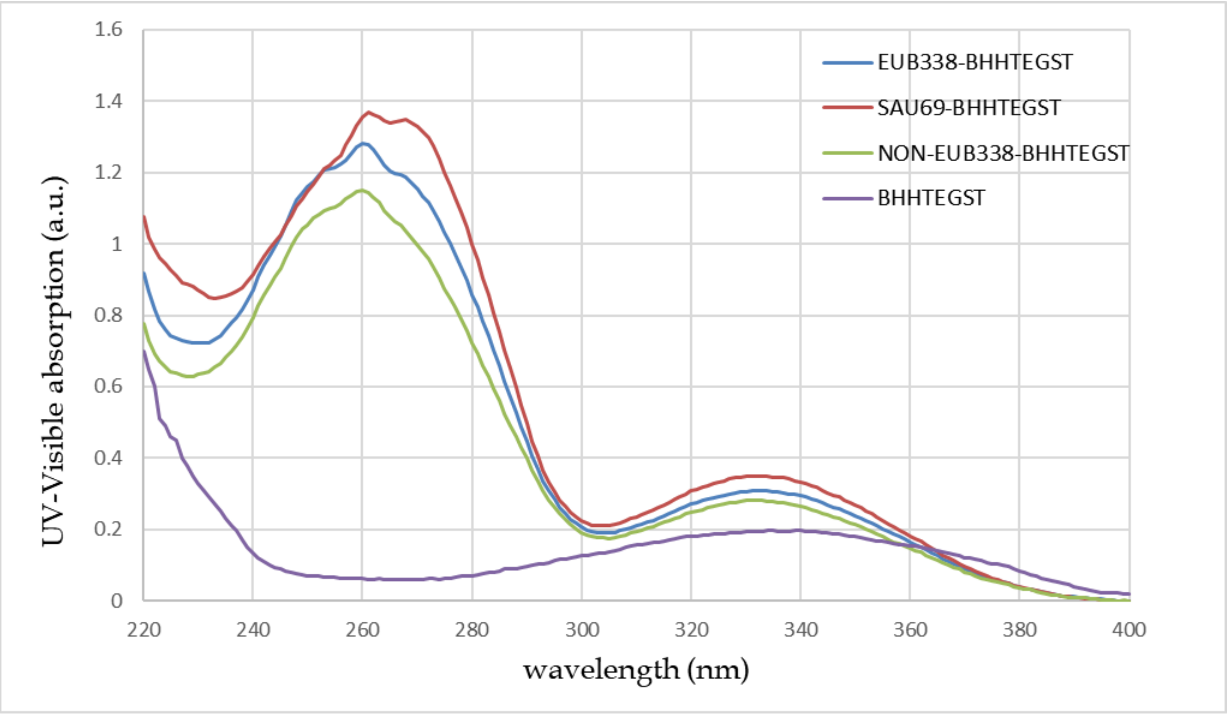

Figure S10: UV-visible absorption spectra for the BHHTEGST ligand and conjugated oligonucleotide probes (~70 µM) (BHHTEGST $\lambda_{ex.max}$ =335 nm and oligo at 260 nm).

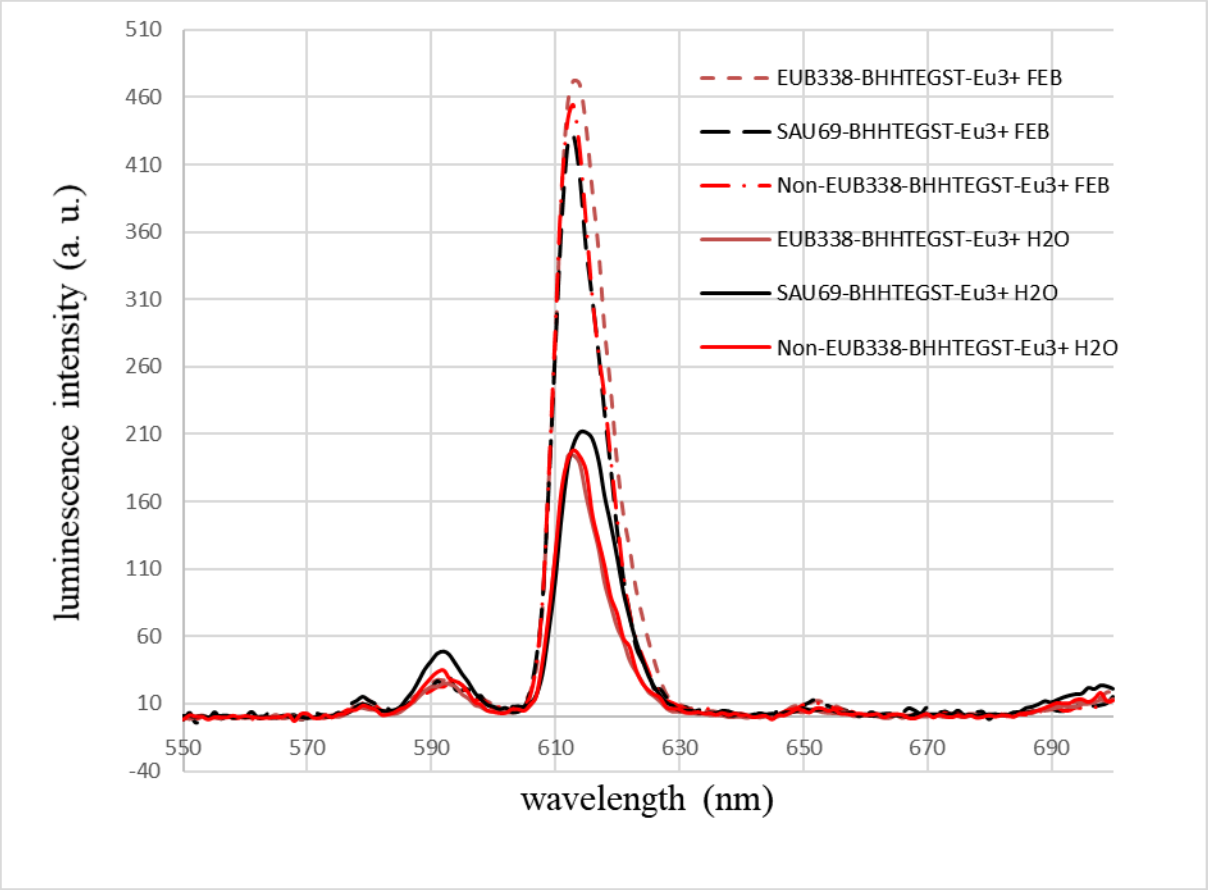

Figure S11: Luminescence emission of oligo conjugates (DNA-BHHTEGST-Eu^{3+} DNA=EUB338, SAU69 and Non-EUB338 (5.0 µM) in FEB and MQ water. Excited at 335 nm and maximum luminescence emission observed at 613 nm.

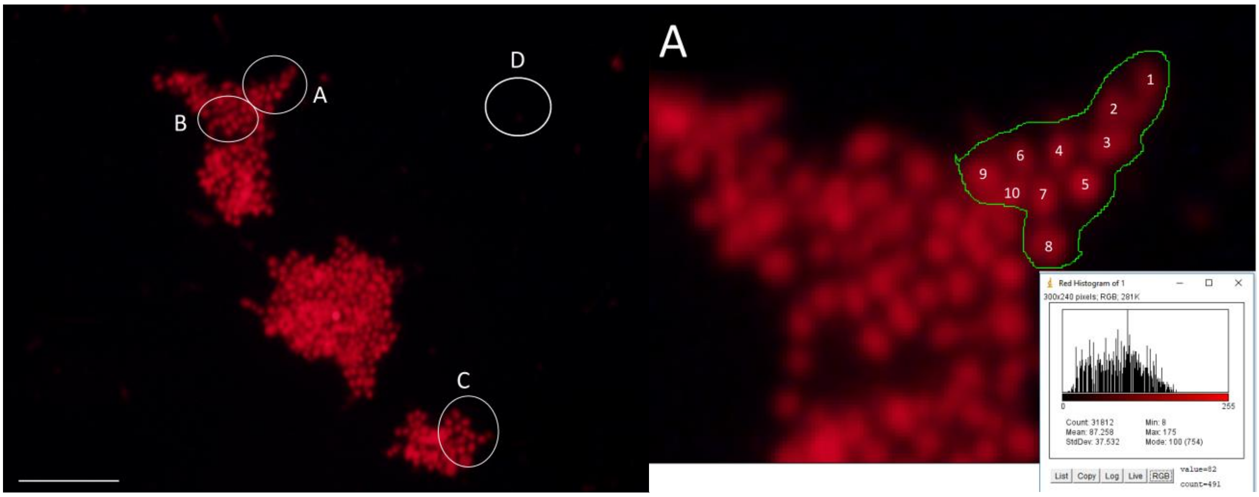

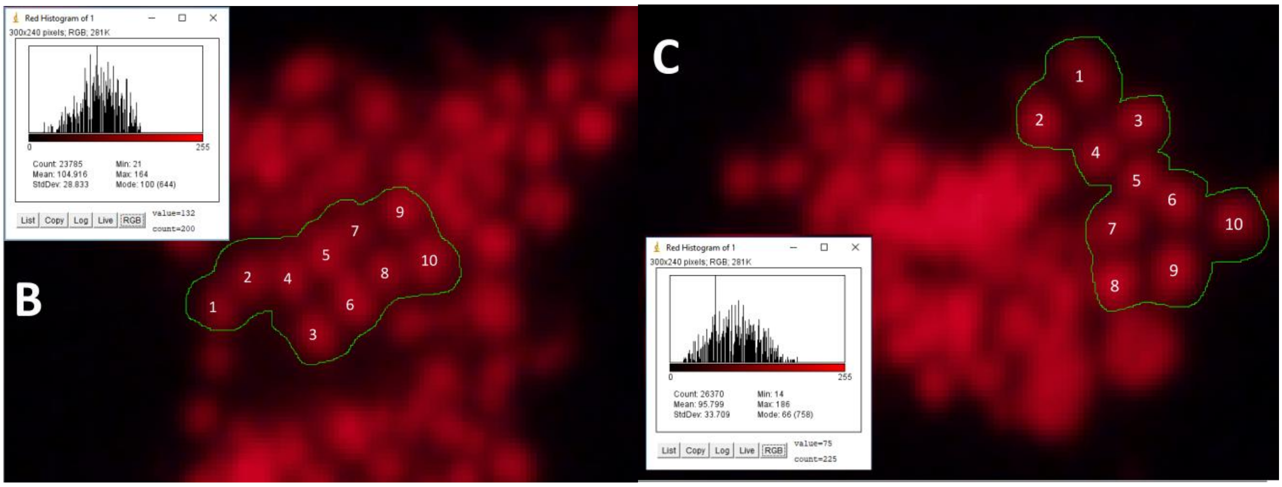

Figure S12. Quantification of the signal-to-noise ratio for the SAU69-BHHTEGST-Eu^{3+} probe LISH labelled ***S. aureus*** using time-gating is shown. The circles (A, B and C) show the regions used for the quantification of the mean signal intensity of the desired target cells and (D) for the background noise; (A, B & C) [zoomed in images] Average mean signal 95.7 [Mean brightness of 10 cells in histogram red channel]; (D) the Background 3.1 [mean brightness of region of background in histogram red channel (not shown)] were measured. The signal-to-noise ratio (SNR) was calculated to be 31.

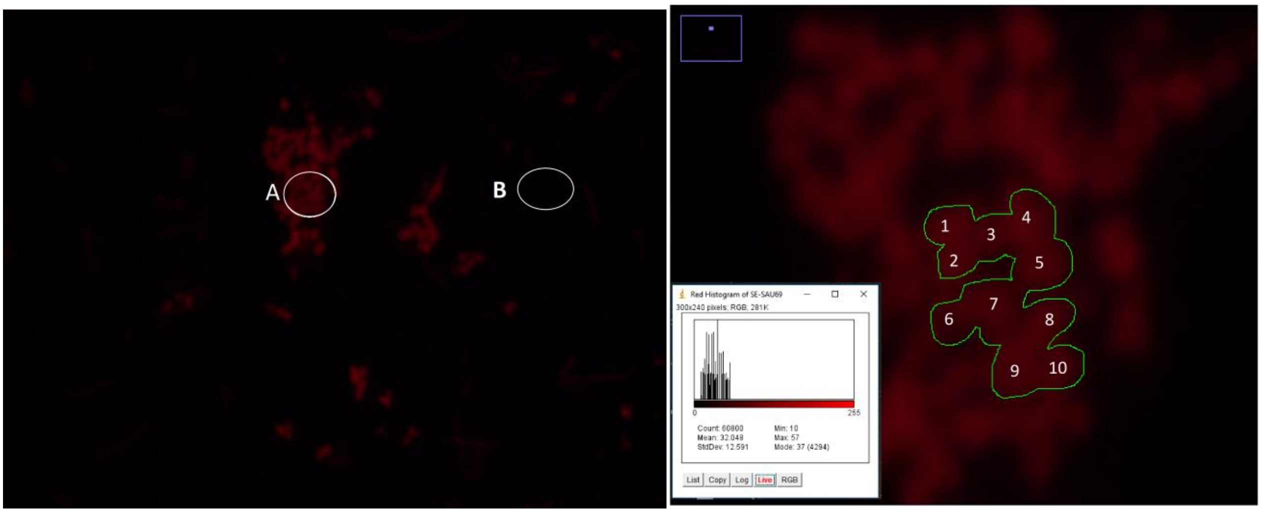

Figure S13. Quantification of the signal-to-noise ratio for the SAU69-BHHTEGST-Eu^{3+} probe LISH labelled *S. epidermidis* using time-gating conditions is shown. The circles (A) show the regions used for the quantification of the mean signal intensity of the desired target cells and (B) for the background noise; (A) [zoomed in images] Mean signal 32 [Mean brightness of 10 cells in histogram red channel]; (B) the Background 6.9 [mean brightness of region of background in histogram red channel (not shown)] were measured. The signal-to-noise ratio (SNR) was calculated to be 4.6.

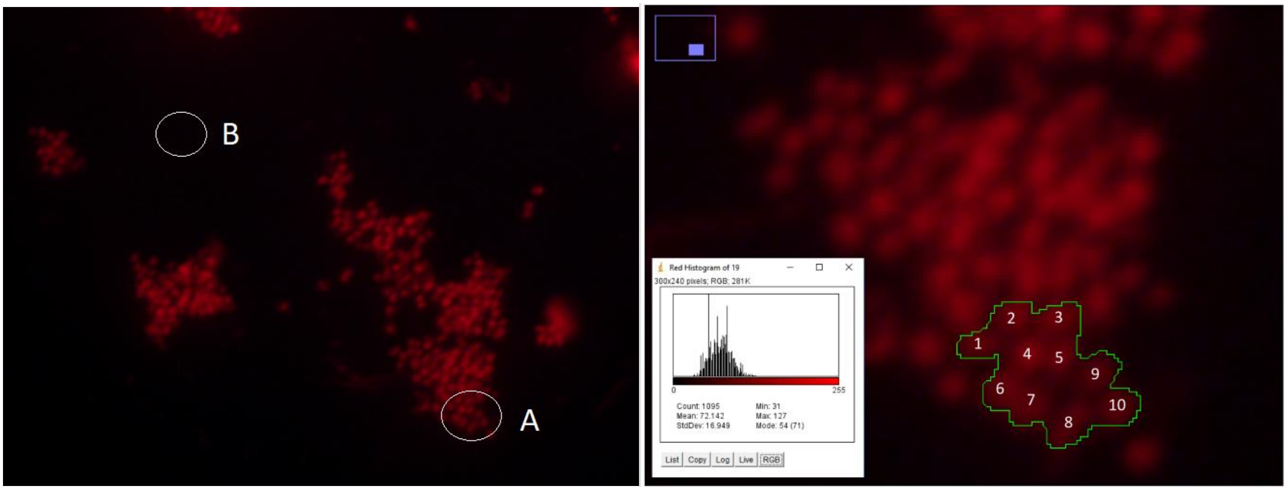

Figure S14. The quantification of the signal-to-noise ratio in EUB338-BHHTEGST-Eu^{3+} probe LISH (Top image) labelled ***S. aureus*** collected under time-gated condition is shown. The circles (A) show the regions used for the quantification of the mean signal intensity of the desired target cells (n=10) and (B) for the background noise; (A) [zoomed in images] Mean signal 72.1 [Mean brightness of 10 cells in histogram red channel]; (B) the Background 5 [mean brightness of region of background in histogram red channel (not shown)] were measured. The signal-to-noise ratio (SNR) was calculated to be 14.4.

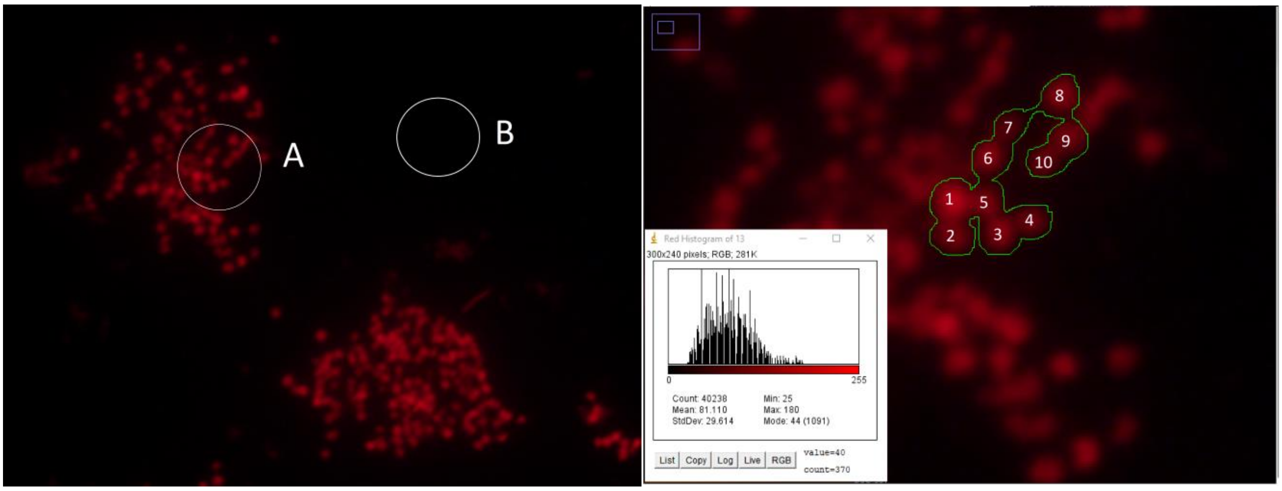

Figure S15. The quantification of the signal-to-noise ratio in EUB338-BHHTEGST-Eu^{3+} probe LISH (Top image) labelled ***S. epidermidis*** collected under time-gated condition is shown. The circles (A) show the regions used for the quantification of the mean signal intensity of the desired target cells (n=10) and (B) for the background noise; (A) [zoomed in images] Mean signal 81 [Mean brightness of 10 cells in histogram red channel]; (B) the Background 5 [mean brightness of region of background in histogram red channel (not shown)] were measured. The signal-to-noise ratio (SNR) was calculated to be 16.2.

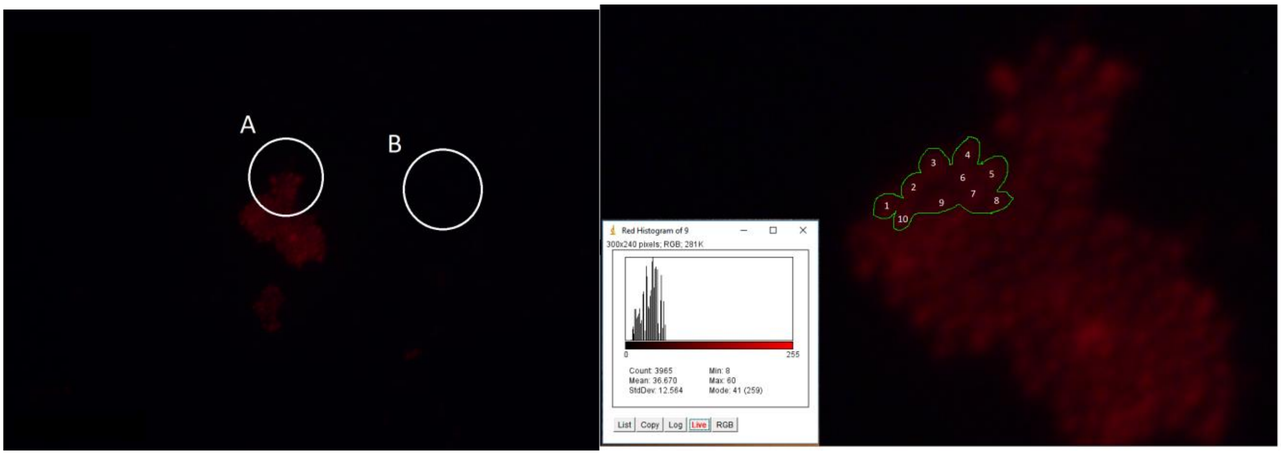

Figure S16. The quantification of the signal-to-noise ratio in Non-EUB338-BHHTEGST-Eu^{3+} probe LISH (Top image) labelled ***S. aureus*** collected under time-gated condition is shown. The circles (A) show the regions used for the quantification of the mean signal intensity of the desired target cells (n=10) and (B) for the background noise; (A) [zoomed in images] Mean signal 36.7 [Mean brightness of 10 cells in histogram red channel]; (B) the Background 6.1 [mean brightness of region of background in histogram red channel (not shown)] were measured. The signal-to-noise ratio (SNR) was calculated to be 6.

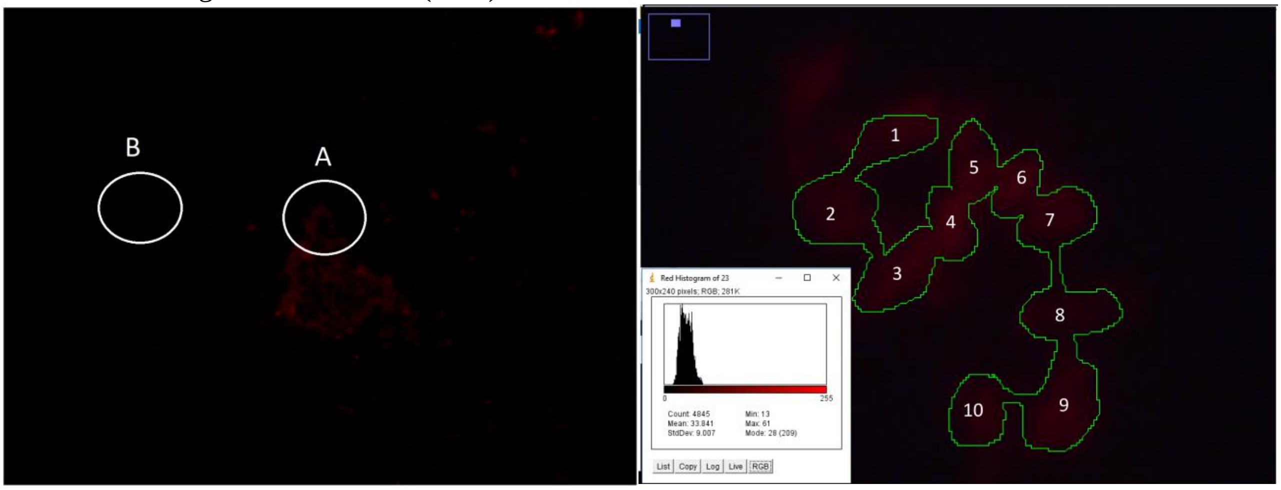

Figure S17. The quantification of the signal-to-noise ratio in EUB338-BHHTEGST-Eu^{3+} probe LISH (Top image) labelled ***S. epidermidis*** collected under time-gated condition is shown. The circles (A) show the regions used for the quantification of the mean signal intensity of the desired target cells (n=10) and (B) for the background noise; (A) [zoomed in images] Mean signal 33.8 [Mean brightness of 10 cells in histogram red channel]; (B) the Background 7.3 [mean brightness of region of background in histogram red channel (not shown)] were measured. The signal-to-noise ratio (SNR) was calculated to be 4.6.

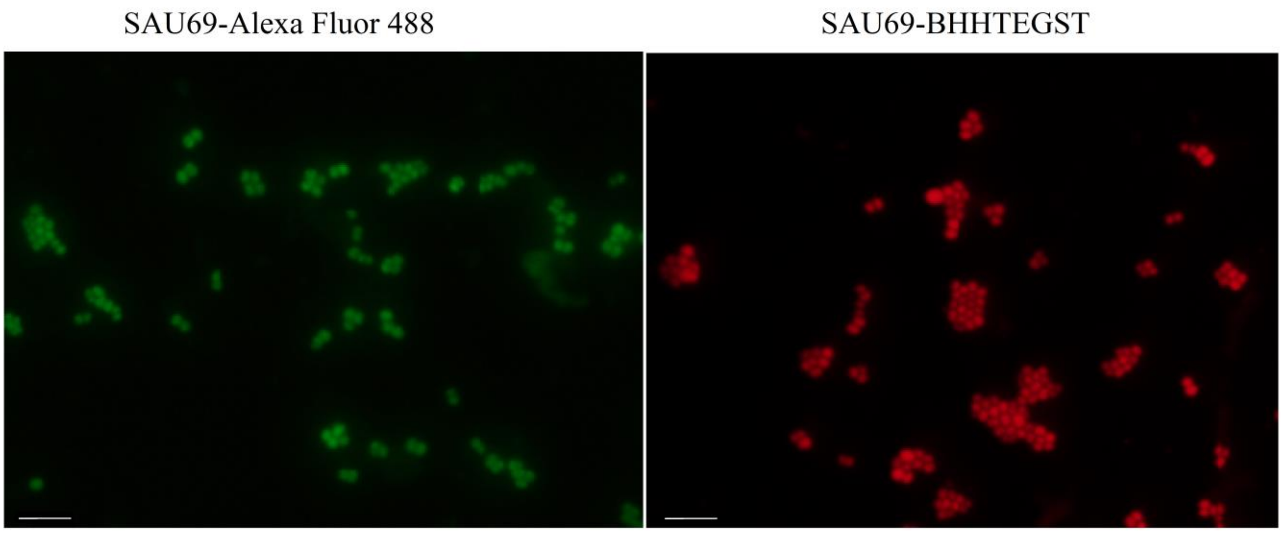

Figure S18. Staining of *S. aureus* cells with SAU69-Alexa Fluor 488 probe (left)and time gated luminescence (TGL) imaging SAU69-BHHTEGSTstaining (right). Scale bar 5 μm.

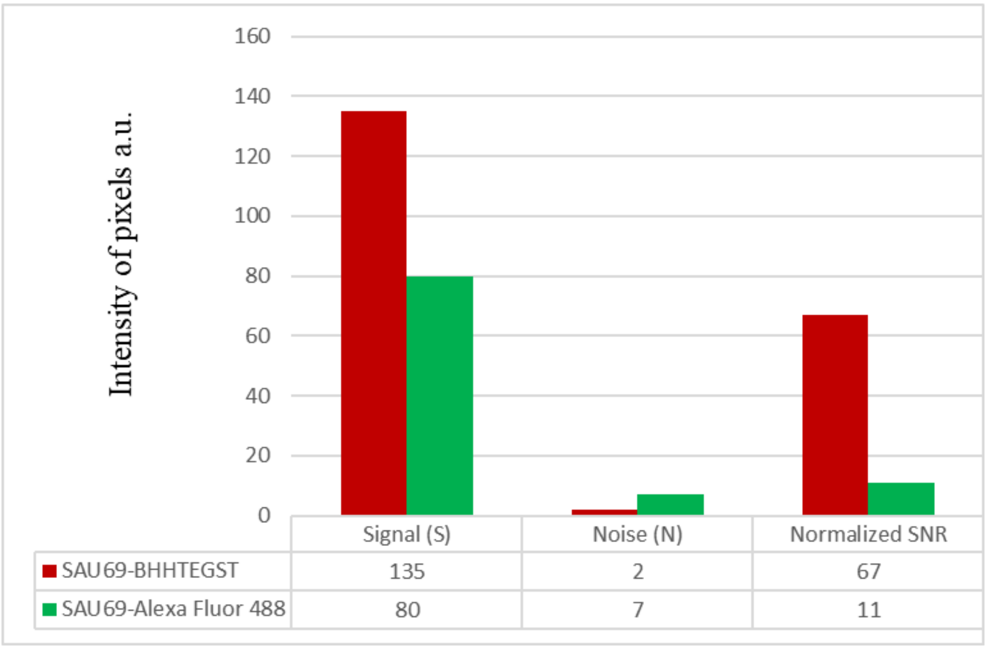

Figure S19. Representation of signal-to-noise ratios (SNRs) for ***S. aureus*** cancer cells labeled with SAU69-BHHTEGST and SAU69-Alexa Fluor 488.

Table S1: Results of images in Figure **S10** to **S15**; The mean signal and background staining of ***S. aureus*** and ***S. epidermis*** are shown. These were labelled with SAU69-BHHTEGST-Eu^{3+}, EUB338-BHHTEGST-Eu^{3+} & NON-EUB338-BHHTEGST-Eu^{3+} probes.

	S. aureus			*S. epidermidis*		
Probe	***Mean Signal***	***Mean Background***	***SNR***	***Mean Signal***	***Mean Background***	***SNR***
SAU69	***95.7***	3.1	31	***32***	6.9	4.6
EUB338	***72.1***	5	14.4	***81***	5	16.2
NONEUB338	***36.7***	6.1	6	***33.8***	7.3	4.6

Table S2: Averaged results of replicated experiments ($n_{\text{replication}}$=30) shown in Table S2: The mean signal and background staining of *S. aureus* and *S. epidermis* are shown. The standard deviation (SDV) was calculated and is also listed.

	S. aureus				*S. epidermidis*			
Probe	***Mean Signal***	***Mean Background***	***SNR***	***SDV***	***Mean Signal***	***Mean Background***	***SNR***	***SDV***
SAU69	***105***	3	35	3.3	***31***	7	4.4	6.9
EUB338	***75***	5	15	4.6	***80***	5	16	5.7
NONEUB338	***30***	6	5	4.4	***27***	7	3.8	4.9
